# Improving Regions of Interest Multivariate Curve Resolution: Development of an Empirical Metric System through the Study of Passive Sampling Extracts of Wastewater in Antarctica

**DOI:** 10.1021/acs.analchem.5c00777

**Published:** 2025-06-13

**Authors:** Barbara Benedetti, Carlos Perez-Lopez, Henry MacKeown, Emanuele Magi, Roma Tauler

**Affiliations:** 1 Department of Chemistry and Industrial Chemistry, University of Genoa, Genoa 16146, Italy; 2 Department of Environmental Chemistry, 203229 Institute of Environmental Assessment and Water Studies (IDAEA-CSIC), Barcelona 08034, Spain

## Abstract

The huge potential of liquid chromatography–high-resolution mass spectrometry (LC-HRMS) still comes along with the challenges of data analysis. Regions of interest multivariate curve resolution (ROIMCR) is a valid chemometric tool when working in data-independent acquisition (DIA), since it provides a link between precursor and product ions based on chromatographic and spectral profiles. Still, the quality of the ROIMCR models should be carefully evaluated for a consequent reliable annotation of non-target chemicals. The present case study deals with the non-target analysis of extracts coming from passive samplers deployed in a wastewater treatment facility in Antarctica (Italian Research Station). The extracts, derived from polar organic chemical integrative samplers (POCIS), were analyzed by LC-DIA-HRMS/MS, resulting in a rich and complex data set. The use of a fit-for-purpose ROIMCR workflow ended in six models for a total of 770 resolved components; among them, approximately 100 compounds were tentatively identified thanks to the recently developed MSident software, including pharmaceuticals and natural substances. The chemical meaningfulness of all resolved MCR components was carefully checked and rationalized for the first time in a classification system, with 7 classes divided into 3 “goodness levels” (A, B, and C). Level A components were characterized by single chromatographic peaks and mass spectra with a reasonable appearance of precursor and product ions. Level B components presented flaws or anomalies in either the chromatographic or spectral profile, and level C components clearly showed unacceptable features. The percentage of high-quality MCR components (level A) ranged from 15 to 48%, while components of acceptable quality (levels A and B) reached percentages between 65% and 85%. Most annotated compounds were indeed associated with good-quality MCR components. The automatization of the proposed classification system may constitute a powerful additional tool to evaluate MCR models’ quality and thus improve the reliability of ROIMCR results when applied to challenging case studies.

## Graphical abstract

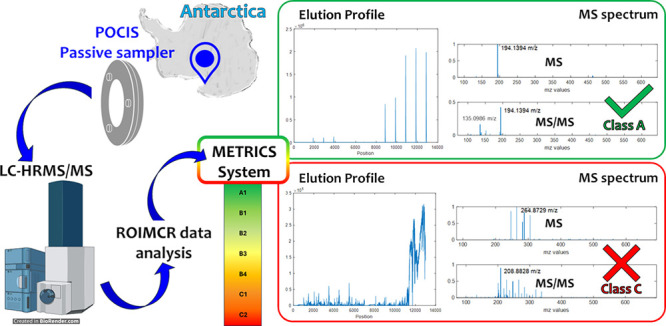

## Introduction

The strength and possibilities of liquid chromatography coupled to high-resolution mass spectrometry (LC-HRMS) are gaining much attention in analytical chemistry. Besides the most common applications in the omics sciences,^1^ environmental studies based on HRMS non-targeted analysis (NTA) are also spreading.^2^

The main goal of NTA is the comprehensive determination of the chemical constituents of a sample, but it suffers from issues related to chemical coverage and quantitative analysis. In fact, the effort to obtain holistic NTA methods is hindered by the complexity of sample composition and the consequent required analytical efforts and workflow.^3,4^ Nonetheless, NTA in environmental sciences is a promising approach from a qualitative point of view, especially for prioritizing pollutants based on their frequency of detection, for its usefulness in discriminating sample groups and for the provided semiquantitative information.^5^

In general, works based on NTA either exploit well-established sample pretreatments or direct injection of liquid samples in the LC system, when possible.^2,6^ Passive sampling is a less used approach, but it still has great potential. When using most passive samplers, accurate quantitative analysis is challenging, due to the necessity of their calibration and the susceptibility to the site conditions.^7,8^ On the other hand, qualitative analysis, as well as comparative semiquantitative analysis, can be easier and more advantageous.^9,10^ Indeed, the ability of passive sampling to sorb and preconcentrate chemical contaminants in situ for several days facilitates achieving lower limits of detection for the substances bound to the sampler compartment(s).^9,11^ This peculiarity makes the combination of passive sampling and LC-HRMS NTA a promising strategy for detecting a wide range of chemicals. Compared to the NTA of classical spot samples, relatively few works have dealt with the combination of passive sampling and NTA.^2,11^ Usually, rich extracts are obtained from passive samplers, resulting from days or weeks of chemicals’ accumulation in the device. These samples thus represent a goldmine of information for NTA, but due to sample complexity, the choice of the most appropriate LC-HRMS method and data analysis should be carefully addressed.

Data processing and compound identification still represent challenges for NTA approaches in general. Several studies have already focused on the NTA data analysis workflow, for an effective prioritization and identification of suspect or unknown contaminants.^12–15^ Normally, these workflows include the following main steps: chromatogram alignment, signal filtering, blank subtraction, feature finding and filtering, peak modeling, formula annotation and structure proposal, aided by databases and libraries.^16,17^ It must be underlined that the most adequate data processing method depends on the chosen acquisition mode. For example, the direct comparison with spectral libraries for compound identification is easier when data-dependent acquisition (DDA) mode is used, with the straightforward association of the tandem mass spectrum (MS2) to a known specific precursor ion (in MS1).^18,19^ A more challenging situation is related to the use of data-independent acquisition (DIA) mode, where all the ionized species at a certain acquisition time are fragmented, generating tandem mass spectra which include product ions of all the precursors.^20,21^ No filter based on the signal intensity or on its identity is applied during the HRMS/MS data collection, theoretically allowing the unbiased detection of less intense peaks. However, the main DIA drawback is that the assignment of product ions to a precursor is not directly feasible. This can complicate the consequent spectral interpretation, also highlighting the importance of an efficient chromatographic separation. Various approaches have been developed to cope with the difficult DIA data processing and interpretation, such as MS-DIAL^22^ and XCMS.^23^ An alternative to those approaches is represented by the ROIMCR strategy, which is based on the simultaneous processing of all the MS data signals, without prior chromatogram alignment and peak shaping.^24^ This chemometric tool has recently proven to be successful in analyzing LC-HRMS/MS data obtained by DIA.^25^ In fact, it allows the direct resolution of the chemical components (componentization) in a set of samples, based on both their chromatographic and spectrum profiles, exploiting the intrinsic bilinear data structure of the LC-HRMS/MS data.

In the present work, LC-HRMS/MS has been used for the analysis of extracts derived from passive samplers (in particular, Polar Organic Chemical Integrative Samplers-POCIS), employing DIA as acquisition mode and ROIMCR for data analysis. The POCIS were deployed in Antarctica, at the outlet of the wastewater treatment facility of the Italian Research Station Mario Zucchelli. These samples, object of a recent work focused on the determination of targeted emerging contaminants,^26^ were characterized by a complex composition, thus representing an interesting case study to verify the potentialities of ROIMCR. The application of ROIMCR served for chemical annotation and multivariate comparison among samples coming from two different campaigns. Moreover, the different levels of complexity of the ROIMCR models permitted the rationalization of a classification system for the MCR resolved components, which is herein proposed for the first time.

## Materials and Methods

### Chemicals and Reagents

An analytical standard mix was injected in each analysis batch, including compounds considered in a previous study as the target analytes (pharmaceuticals, personal care products, plasticizers, UV-filters, and tracers). Details on the complete list, their purity and suppliers are provided in section S1 of Supporting Information. Mass grade MeOH, acetonitrile (ACN), formic acid, and ultrapure water were purchased from Merck (Darmstadt, Germany).

### Samples and Sample Treatment

Passive sampling was carried out using commercial POCIS with hydrophilic lipophilic balanced (HLB) sorbent, purchased from E&H services (Prague, Czech Republic). A full description of the sampling strategy, the sampler configuration and the POCIS processing is reported in MacKeown et al., 2024.^26^ Passive samplers were deployed at the outlet of the wastewater treatment facility of the Mario Zucchelli Antarctic research station (latitude, −74° 41′ 36.95″ S; longitude, 164° 06′ 42.12″ E). Two Antarctic sampling campaigns were considered, performed in the period November 2021 to February 2022 (sampling campaign 1, c1) and November 2022 to January 2023 (sampling campaign 2, c2). Six and five deployments of 2 weeks were performed in the first and second campaign, respectively. The samplers were stored frozen until extraction.^26^ After POCIS processing, the concentrated eluates were diluted 100-fold with MeOH:water, 1:1 (v/v), and filtered before analysis.

The sample set analyzed by LC-HRMS/MS thus consisted of 11 POCIS extracts (details in Table S1), one procedural blank for each campaign (unexposed POCIS), and one standard mix.

### Instrumental Analysis

The instrument used for the analysis was an ultrahigh performance liquid chromatograph (UHPLC) coupled to a quadrupole-time-of-flight mass spectrometer (Impact II Q-ToF) through a heated electrospray ionization source (HESI), from Bruker (Billerica, Massachusetts, United States). The chromatographic separation was achieved by a pentafluorophenyl (PFP) core–shell column (100 mm × 2.1 mm i.d., particles diameter 2.6 μm) by Phenomenex (Torrance, USA). Two separate analyses, in ESI positive and negative acquisition modes, respectively, were performed for each sample. The mobile phases were water (phase A) and ACN (phase B), neutral for the analysis in negative ionization mode and with the addition of 0.1% Formic acid for those in positive ionization mode. In both cases, a gradient elution of 46 min (including the re-equilibration time) was used. Mass spectra were acquired by employing DIA, with a nominal resolution of 60 000. The simple MS data (defined as MS1) were acquired in full scan with *m*/*z* in the range 60–800 Da, and the tandem mass spectra (defined as MS2) were acquired by applying a ramp of collision energy (24–36 eV), to generate fragments of all the ions detected in MS1. More details on the LC-HRMS/MS method are presented in Supporting Information (section S2 and Table S2).

### Application of ROIMCR

Figure 1 depicts the workflow followed to apply ROIMCR, which implies the combination of the regions of interest (ROI^27,28^ ) and the multivariate curve resolution-alternating least squares (MCR-ALS^29,30^ ) computational methods. Figure 1 displays the ROIMCR workflow for the positive acquisition mode; the same was applied to the data obtained by DIA in the negative ionization mode. Details on the theory of the ROIMCR model can be found in previous papers.^24,31^ All of the data processing was performed in the MATLAB environment (release 2023b, The MathWorks, Inc., Natick, MA, USA).

**Figure 1:**
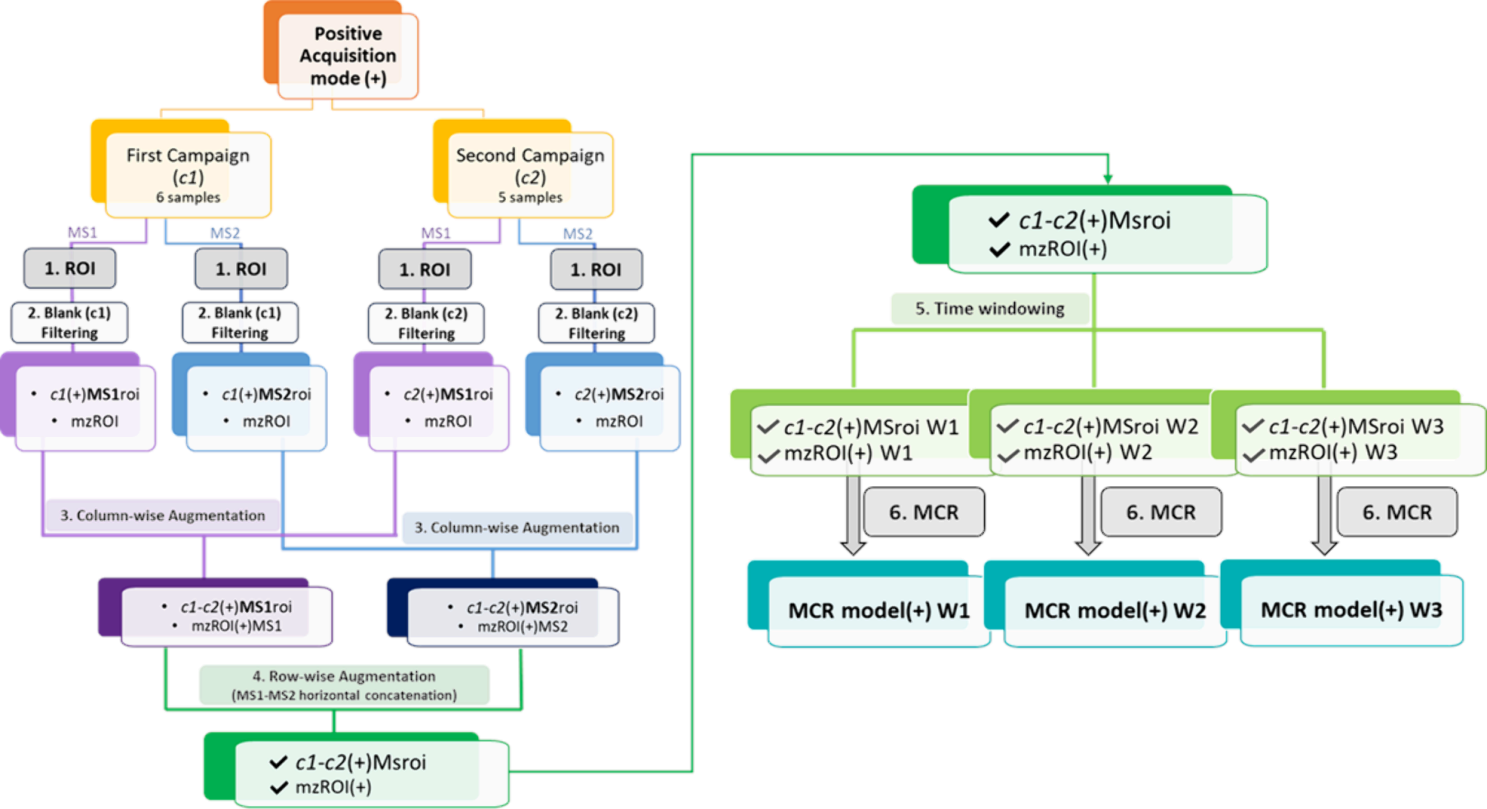
ROIMCR workflow for the data obtained from the ESI positive ionization mode (indicated with the symbol “(+)”). The same workflow was applied for the data acquired in ESI negative ionization mode (matrices will be indicated by the symbol “(−)” in the text).

### ROI and Blank Subtraction for Each Campaign

The ROI algorithm was applied to the two groups of samples (campaigns) separately, to correctly subtract the procedural blank signals of each group. The ROI graphical user interface (GUI) software program was used^28^ to extract the ROI signals of the two groups (step 1 in Figure 1). The ROI compression procedure was separately applied to MS1 and MS2 for each sampling campaign. MS1 and MS2 signals were selected using the following parameters: elution time range 44–2030 s (min 0.7–33.8) for the positive acquisitions and 44–1696 s (min 0.7–28.3) for the negative acquisitions, to exclude the less informative initial and final parts of the chromatographic runs; *m*/*z* range 80–700 Da; signal intensity threshold of approximately 1% of the most intense MS1 signal and approximately 3 times lower for MS2 signals, in order not to miss low intensity significant fragmented ions; 0.005 Da as accepted instrumental mass accuracy (all *m*/*z* values within this accepted error were grouped as coming from the same MS signal, equal to their mean *m*/*z* value); a minimum number of 6 signal occurrences to depict a good chromatographic peak (considering the fast scanning speed of the Q-ToF mass spectrometer^32^). Therefore, 4 MSroi data matrices with their corresponding *m*/*z* values arrays (mzROI) were obtained for the signals acquired at MS1 and MS2, from the two sample campaigns. A blank subtraction using the blank passive sampler processed for each campaign was performed (step 2 in Figure 1): *m*/*z* signals were maintained only if their intensities in each sample were at least 4 times higher than in the respective blank. Thus, filtered MSroi data matrices enclosing the chromatographic profiles of all ROIs (c1­(+)­MS1roi, c1­(+)­MS2roi, c2­(+)­MS1roi, and c2­(+)­MS2roi in Figure 1), and their corresponding mzROI cleaned arrays, were obtained.

### Building Column- and Row-wise Augmented MSroi Time Window Data Matrices

Column-wise augmented MS1roi matrices were achieved by vertical concatenation of MS1roi data matrices from the different samples analyzed and the same was performed for the MS2roi data matrices (step 3 in Figure 1).^33^ Then, the MS1 and MS2 signals were row-wisely augmented (horizontal concatenation), as indicated in step 4 in Figure 1. In this way, a single column- and row-wisely augmented MSroi data matrix and a mzROI array, which included all the data (MS1 and MS2) of the two campaigns, were created (c1-c2­(+)­MSroi, mzROI­(+) in Figure 1). Due to the large number of ROI signals found (see Table S3), the data matrices for each sample were divided into three time windows prior to the MCR application (step 5 in Figure 1), with some time overlaps among them (as shown in Figures S1 and S2). For positive ionization data, time window W1 corresponded to the time interval 44–716 s (min 1–11.93); time window W2 corresponded to the time interval 582–1255 s (min 9.7–20.9), and time window W3 corresponded to the time interval 1121–2030 s (min 18.7–33.8). For the negative ionization data, time windows W1–W3 corresponded to the time intervals 44–718 s (min 0.7–11.96), 584–1257 s (min 9.7–20.9), and 1123–1696 s (min 18.7–28.3), respectively. The three time window data matrices of every sample were then concatenated vertically to give three new column-wise augmented time window data matrices (*c1-c2*(+)­MSroi W1, *c1-c2*(+)­MSroi W2, *c1-c2*(+)­MSroi W3; see step 5 in Figure 1) with their associated mzROI arrays.

As initially mentioned, the whole workflow depicted in Figure 1 was repeated for the data acquired in negative ionization mode (indicated with the symbol “(−)” in the following paragraphs).

### MCR-ALS of the Different Time Window MSroi Data Matrices

MCR-ALS analysis (step 6 in Figure 1) of MSroi data matrices performs their bilinear model non-negative factor decomposition,^29,30^ giving the chromatographic elution and MS spectra profiles of the components present in the analyzed samples (see Dalmau et al.^31^ for details on the application of MCR-ALS to LC-MS data). MCR-ALS was applied to the three time window MSroi augmented data matrices in positive and negative acquisition modes, using the MCR GUI software.^30^ One MCR model was computed for each time window by manually setting the number of components in the range 60–190 (depending on the time window and on the MS ionization mode). The singular value decomposition (SVD) of the MSroi data matrices gave a first estimation of the number of components needed to explain the data variance.^31,34^ Models with a different number of components were tested to check for a significant improvement in the percentage of explained variance and to recover reliable elution and spectra profiles.^24^ To initiate the ALS optimization algorithm, the most different elution profiles (at the “purest” *m*/*z* variables; see ref 35) were used as a first estimation. Non-negativity constraints were applied to both MCR elution profiles and MCR resolved spectra, together with the spectra normalization to equal height to avoid scale resolution ambiguity and have the most intense signals of all MCR resolved spectra equal to 1. MCR-ALS outputs were the chromatographic elution (**C**) and mass spectra (**S** ^**T**^) profile matrices of the MCR resolved components, as well as the percentage of explained data variances (*R* ^2^) and lack of fit for each model.^30,36^

### MSident

MS spectra of precursor ion (MS1) and fragments (MS2) were directly linked in every MCR resolved component, allowing their chemical identification by the MSident tool,^37^ by comparison with those available in spectral libraries. The databases Human Metabolome Database (HMDB), MassBank, MassBank of North America, and MSdial were used. The parameters set in the MSident software GUI were the following: exclusion of normalized MS signals lower than 0.1 (10% of maximum intensity); precursor ions normalized MS1 signal intensity equal to or higher than 0.4; maximum difference of ROI *m*/*z* values (between experimental and library spectra) of 10 ppm in MS1 and 20 ppm in MS2; score identification thresholds higher than 500. For more details see previous work.^37^

### Further Chemometric Data Analysis

Once the elution profiles of the different components were resolved, their peak areas were calculated and used to obtain a multivariate comparison between the two campaigns. Principal component analysis (PCA) was applied to the elution profile peak areas of negative and positive ionization data, separately. PCA was applied after data autoscaling using a script created in the MATLAB environment (release 2023b, The MathWorks, Inc., Natick, MA, USA).

## Results and Discussion

### Extraction of the MS Regions of Interest

The deployment of POCIS at the outlet of the Antarctic wastewater treatment facility for 2 weeks allowed the accumulation and preconcentration of organic compounds.^38^ As a result, low-concentration emerging contaminants were sampled along with other major chemical compounds present in the effluent waters, resulting in rather complex sample extracts.

In a previous study, several analytes, including caffeine, anti-inflammatory drugs and UV filters, were quantified by targeted LC-MS/MS analysis in the same samples.^26^ These results were exploited to select the most appropriate threshold values of signal intensities for the implementation of the ROI algorithm, allowing the identification of a large number of significant *m*/*z* values, while avoiding those related to noise. In particular, the intensity threshold values for the MS1 profiles were set at a value that guaranteed the detection of the *m*/*z* of some contaminants known to be present at significant concentrations in the analyzed samples, namely, caffeine, theobromine, and nicotine. This value was approximately 1% of that of the most intense MS signal acquired. Noise and interferent signals were mostly eliminated by using blank subtraction. Approximately 10% of the MS1 signals were removed, while for MS2, around 20% were eliminated. The number of *m*/*z* values (columns) finally selected for the different MSroi data matrices varied from approximately 800 to 2500. Table S3 gives the number of ROIs of the analysis of the individual and augmented data matrices. Common ROIs were encountered in the data sets from the two sampling campaigns, suggesting a partial overlap in the composition of the treated wastewater in the two years.

### Application of MCR-ALS to Negative and Positive Acquisition Data Matrices

At first, MCR-ALS was applied to the whole chromatographic profile and to a different number of time windows; the results of these preliminary tests are reported in the Supporting Information (section S3). The time-windowing of the LC-MS/MS chromatographic profiles allowed reducing the MCR-ALS computation times and facilitated their accurate processing.^39,40^ Indeed, the presence of many low MS signals represented a challenge because these contributions could be considered as noise and/or be embedded in more intense signals.

Table 1 summarizes the results of the componentization of the three time windows in both ionization modes. For positive ionization data, in the MCR-ALS analysis of time windows W1, W2, and W3, 60, 180, and 140 components were selected as optimal, respectively. Time window W3 was the most difficult to analyze, due to the presence of a higher background signal contribution and more interferent peaks (strongly retained impurities eluted at higher percentages of organic solvent).^41^ Some examples of the obtained MCR elution and spectral profiles are shown in Figure S3.

**Table 1:** MCR Fitting Results and Number of Components Selected for the Whole Sample Set

MCR model	number of MCR components	*R*^2^^a^ (%)	Lof^b^ (%)
Model (+) W1	60	98.24	13.3
Model (+) W2	180	97.11	17.0
Model (+) W3	140	95.02	22.3
Model (−) W1	100	97.96	14.3
Model (−) W2	190	98.22	13.4
Model (−) W3	100	98.63	11.7

a % of variance explained by the model.

b % of lack of fit: parameter related to the residuals not explained by the MCR model (the lower the better).^29^

For the data obtained by negative acquisition mode, overall good data fitting was obtained in the MCR-ALS analysis of the three time window data sets, with 100, 190, and 100 components, respectively. Two examples of satisfactorily resolved MCR elution and spectral profiles for the negative ionization data are shown in Figure S4. In all cases, the percentages of explained variance were between 95% and 99% and the lack of fit was between 11% and 22%.

### Classification of MCR Resolved Components

After the application of MCR-ALS, the “reliability” of the components was verified. Indeed, the number of components should not be taken as the exact number of compounds detected in the samples. Considering the chemical context of LC-HRMS/MS analysis, an MCR component is either accepted or not as associated with a particular chemical depending on (i) its resolved chromatographic profile (peak shape) and (ii) the appearance and signals of its MS spectrum (both in MS1 and MS2). For this reason, each MCR model was carefully evaluated, by visually checking if the individual resolved component profiles (elution and spectrum) could be associated with a chemical species without ambiguities.^40^ As a result of this evaluation, a classification system of the MCR components is proposed based on different levels of reliability from a chemical point of view. Seven classes of MCR components were hypothesized, grouped by their quality level, as described in Table 2. Further details on the characteristics of the different classes are given in the Supporting Information (section S4).

**Table 2:** Classification System Proposed To Evaluate the Reliability and Goodness of the Resolved MCR Components Obtained by the ROIMCR Modeling

class	quality level	description of the spectral profile	description of the chromatographic profile
A1	High (A)	-A maximum of 3–4 main peaks in the MS1 spectrum are observed (plus possible isotopic peaks), with *m*/*z* values attributable to common adducts.	A single peak is observed, with Gaussian shape and signal-to-noise ratio >10.
		-The peaks in the MS2 spectrum present lower intensities and lower (or equal) *m*/*z* values compared to the base peak in MS1.	
B1	Medium (B)	-The same requirements as for class A are fulfilled with respect to the MS1 spectrum.	A single peak is observed, with Gaussian shape and signal-to-noise ratio >10.
		-The peaks in the MS2 spectrum present a relative abundance below 5% compared to the base peak in the MS1 spectrum (probable noise signals).	
B2	Medium (B)	-A maximum of 3–4 main peaks are observed (plus possible isotopic peaks) in the MS1 spectrum; other peaks have a relative abundance below 10% compared to the MS1 base peak (MS1 noise peaks).	A single main peak is observed, with Gaussian shape and signal-to-noise ratio >10. Secondary peaks with lower signal-to-noise ratios and/or not perfectly Gaussian shape (noise peaks) are observed.
		-The peaks in the MS2 spectrum present lower intensities and lower (or equal) *m*/*z* values compared to the base peak in MS1; other peaks have a relative abundance below 10% compared to the MS2 base peak (MS2 noise peaks).	
B3a	Medium (B)	-A maximum of 3–4 main peaks on MS1 are observed (plus possible isotopic peaks) in the MS1 spectrum; MS1 noise peaks are present.	Two or more peaks with Gaussian shape, signal-to-noise ratio >10 and comparable areas are observed; noise peaks may also be present.
		-The peaks in the MS2 spectrum present lower intensities and lower *m*/*z* values than the base peak in MS1; MS2 noise peaks are present.	
B3b	Medium (B)	More than 3–4 peaks (plus isotopic peaks) are observed in the MS1 spectrum and/or their *m*/*z* distance makes them not attributable to adducts nor isotopic peaks; MS1 noise peaks are present.	A single main peak is observed, with Gaussian shape and signal-to-noise ratio >10.
B4	Medium (B)	-More than 3–4 peaks (plus isotopic peaks) are observed in the MS1 spectrum and their *m*/*z* distance makes them not attributable to adducts nor isotopic peaks.	Two or more peaks with Gaussian shape, signal-to-noise ratio >10, and comparable areas are observed.
		-Several MS2 peaks are present, suggesting the fragmentation of more than one compound.	
C1	Low (not acceptable) (C)	-A maximum of 3–4 main peaks on MS1 are observed (plus possible isotopic peaks) in the MS1 spectrum; MS1 noise peaks are present.	A very noisy chromatogram, with one or more peaks with signal-to-noise ratio <10, is observed.
		-One or more peaks in the MS2 spectrum have a *m*/*z* value greater than those of the MS1 peaks.	
C2	Low (not acceptable) (C)	-Too many peaks of similar intensities are present in both the MS1 and MS2 spectrum, easily attributable to noise.	An aberrant and noisy chromatographic profile is observed (no peaks of Gaussian shape).

An example of class A MCR component is shown in Figure 2, while Figure 3 illustrates examples of MCR components belonging to the B and C classes. Figures S5 and S6 show detailed examples of all B and C classes. The good or acceptable quality of elution and spectral profiles (classes A, B1, and B2) made the compound annotation attainable, while the presence of noise signals or the possible incorporation of more than one chemical into a single component (B3a, B3b, and B4 classes) hampered the process.

**Figure 2:**
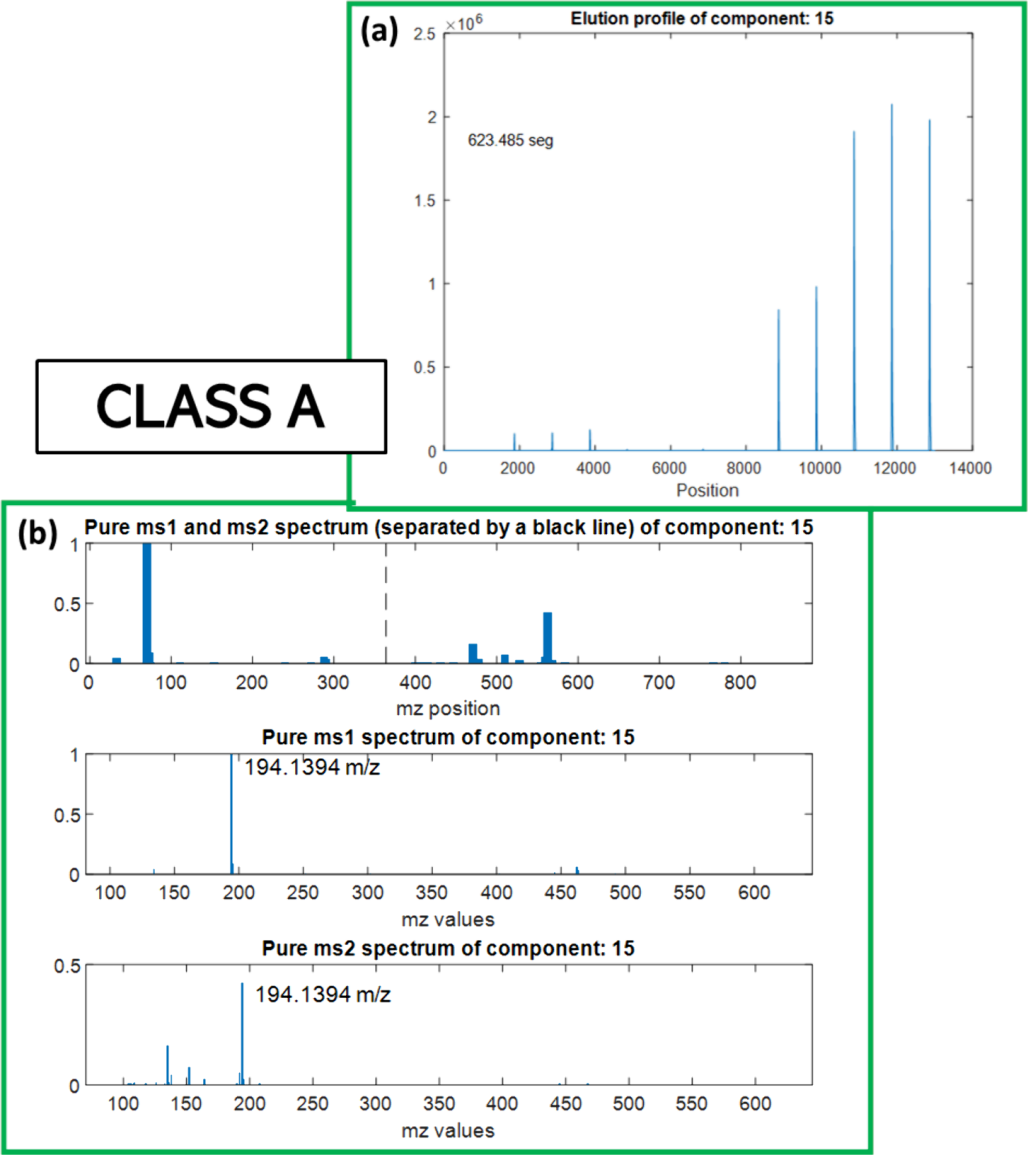
Example of a MCR resolved component classified as A1 (from window W1 of positive acquisition data). In (a), the elution profiles of all analyzed samples are displayed one next to the other (the resolved component was present in 8 samples). The MS1 and MS2 spectra profiles in (b) are displayed together in the upper part and separately below.

**Figure 3:**
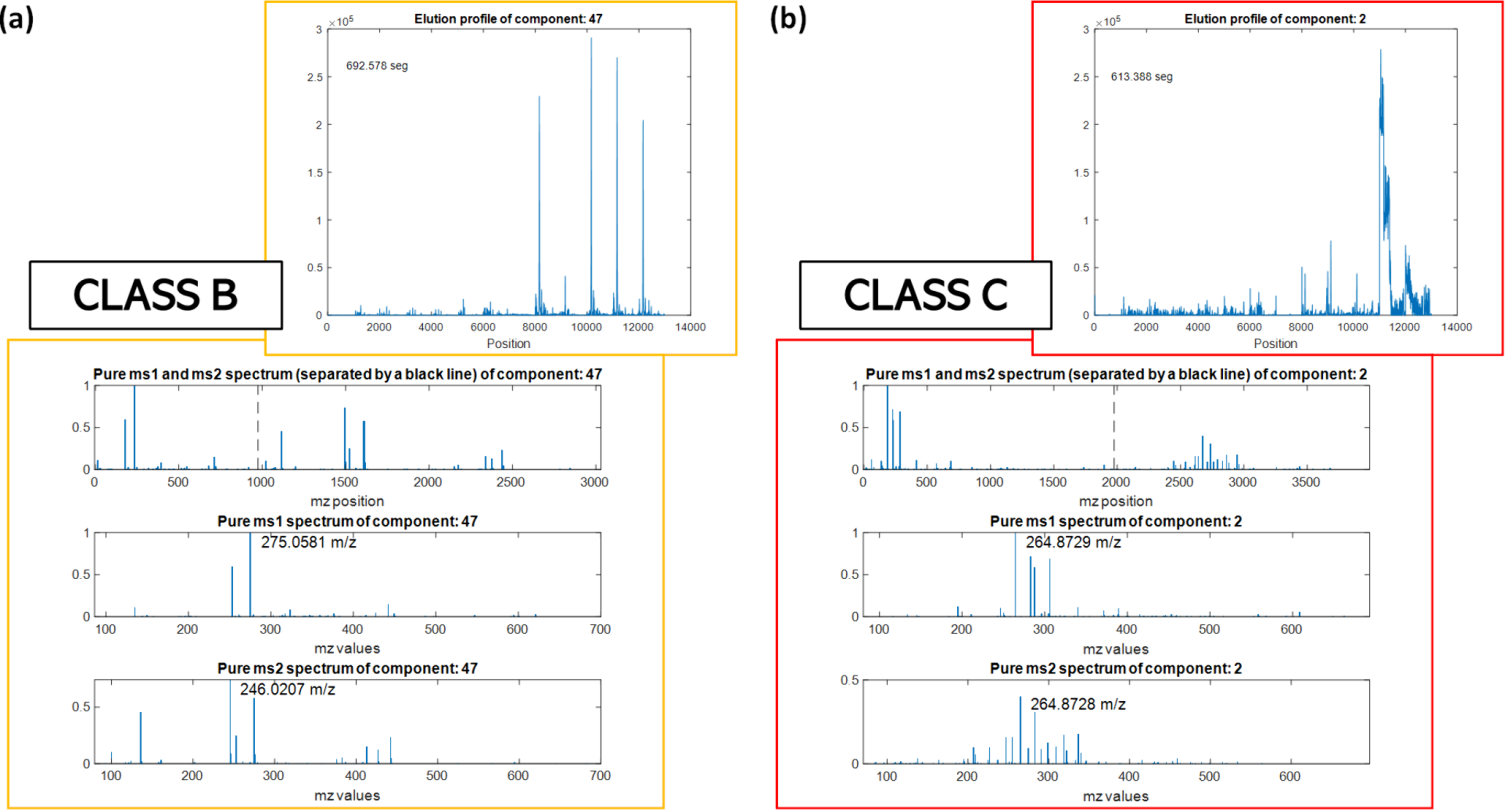
Examples of MCR components classified as B (a) and C (b); elution and spectral profiles are of acceptable and not acceptable quality, respectively, as explained in Table 2. These MCR components were not annotated.

The class assignment of the resolved MCR components showed a different distribution, depending on the chromatogram time window analyzed and the ionization mode. The percentage of components at each goodness level (A, B, or C) is summarized in Figure 4, with the detailed class distribution for two exemplifying time windows. The class distribution of all time windows analyzed is reported in Supporting Information (Figure S7).

**Figure 4:**
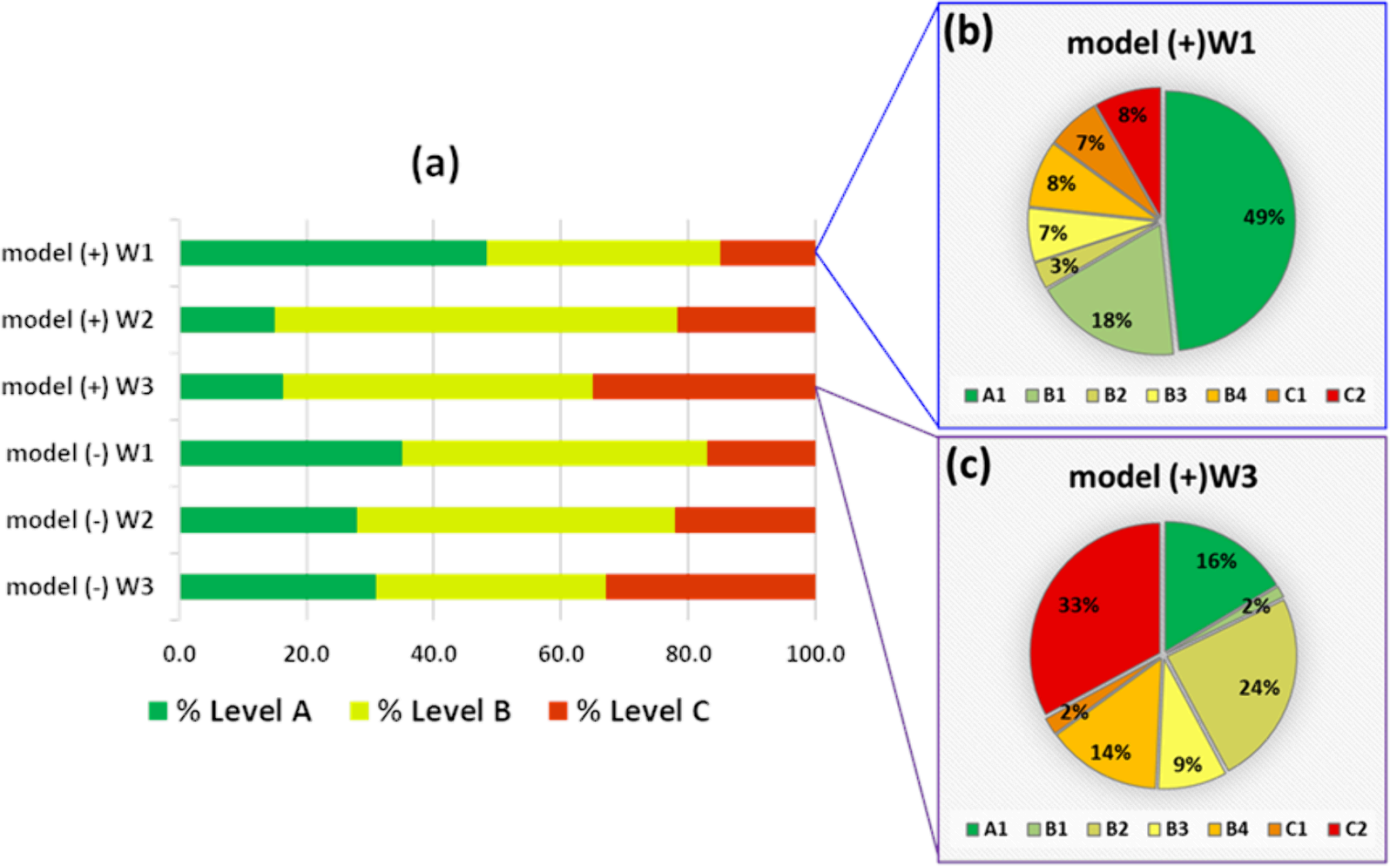
(a) Percentage of MCR components belonging to A, B, and C quality levels of Table 2 (W1, W2, and W3 are the three selected chromatographic time windows, and “+” or “–” their polarity mode acquisition). Detailed distribution of the 7 classes in W1 (b) and W3 (c) time windows in positive ionization mode.

The highest percentage of level A components was found in the first time window (W1) for both ionization modes, suggesting that the lower complexity of the data in W1 (lower number of MCR components) helped to generate more components with high chemical reliability. Generally, the first part of the chromatogram is expected to be “cleaner” (less elution peaks); the central part is characterized by a higher density of significant peaks, while the final part of the chromatographic run is always noisier and has a significant baseline drift. These aspects affected the MCR modeling, with a clear shift toward a worse quality of the resolved MCR components going from W1 to W3. Indeed, the highest percentage of components belonging to C2 class (approximately 30%) was found in the W3 time windows for both ionization modes (see Figure S7). The examples of the component class distributions for W1 and W3 of the positive ionization data (Figure 4) highlight this trend. These results can help in future studies, to select a proper time windowing of the chromatographic runs.

### Identification of MCR Components

Once MCR components were critically evaluated, MSident tool was employed for their chemical annotation. The four databases used gave a total number of 35 and 99 identified components, in positive and negative ionization modes, respectively. Still, some annotations were removed if characterized by precursor ion error >5 ppm, score <700, and MCR class ≠ A, while those related to class C components were considered with caution, reducing the number of more reliable identifications to 20 and 80. The complete list of annotated compounds is given in Table S7 of Supporting Information), together with their corresponding matching scores, statistical relevance, and additional chemical information. For some MCR components, more than one chemical species was matched. This may be ascribed both to the presence of structural isomers and/or to the ambiguities in the MS2 signals assignation. Although more components were identified in the negative acquisition mode compared to the positive one, in the negative mode several components had the same base peak in their MS1 and MS2 spectra. This causes a lower confidence level in their chemical identification (similar to level 4 according to Schymanski et al.^42^), due to the absence of diagnostic product ions in the MS2 spectrum; this outcome probably indicates that the potential fragments formed in the collision cell had generated rather low signals, fallen below the ROI threshold value, also due to the lower ionization efficiency in the negative mode.

Among the annotated compounds, caffeine, nicotine, metformin, and atenolol were also detected in the previous targeted study;^26^ thanks to the availability of analytical standards, these compounds could be assigned at confidence level 1.^42^ Some other compounds found in the targeted analysis were not identified in this work, probably due to the lower sensitivity in the DIA non-targeted analysis. Indeed, the ionization parameters and collision energies used in DIA were a compromise for the detection of as many unknown species as possible and not optimized for some specific analytes, as in targeted analysis.

Within the list of annotations, several drugs and their biotransformation metabolites were identified by the non-targeted ROIMCR analysis, such as antivirals, anti-inflammatories, synthetic hormones, and antihistamines, as well as some personal care products (e.g., surfactants). Several endogenous metabolites were also annotated by MSident, such as lipids (including hormones) and amino acids, as well as other natural substances (polyphenols, especially in their glycosylated forms, terpenes, and few mycotoxins).

Finally, some other MCR components, not clearly identified by MSident, were hypothesized based on their spectrum profiles. These were characterized by peaks in their MS1 spectrum being evenly separated, indicating the loss of repetitive chemical units, characteristic of polymeric compounds. An example of such a MCR component is shown in Figure S8, where a repetitive difference of 44 Da between the peaks in its MS1 spectrum was observed, which possibly indicates the presence of polyethylene glycol (PEG) subunits. This compound could be associated with a sample contamination, but its detection in the samples after the procedural blank filtering suggests that the source comes from the environmental samples and it might be related to the use of personal care products.^44^

When ROIMCR was not coupled to MSident, component identification needed a more time-consuming manual comparison with external databases, with a consequently lower efficiency. For example, in a previous work^100^ only 26 lipids, distinguishing among rice samples of different types, were tentatively identified, and in a similar way, in a prostate cancer study, only 7 relevant metabolites, discriminating among urine samples of healthy and ill individuals, could be annotated.^39^ In two recent works combining DIA and ROIMCR, 23 and 17 PFAS were identified in spiked bird egg samples.^25,45^ The annotation of approximately 100 ROIMCR components in this work represents a good achievement, considering the rather limited information available at present in public repositories and the difficulties still related to the processing of DIA data sets. It is worth remembering that the number of identifications depends on the sample complexity, the MS instrumentation and acquisition mode, the input parameters in the ROIMCR application, and the reference spectra databases.

### Multivariate Comparison of the Analyzed Antarctic Samples

Elution peak areas of the different MCR components in the two Antarctic campaigns were additionally subjected to PCA, after removing those classified at quality level C (based on Table 2). So, a total number of 287 and 298 MCR resolved components were considered for the positive and negative data acquisition modes, respectively. As shown in the two PCA biplots in Figure 5a,b, the two first principal components (PC) allowed the distinction between the samples of the two campaigns (c1 and c2). Figures S9 and S10 additionally highlight the separation of samples on the three first PCs, which together accounted for 76.6% of the total data variance. High correlation among the original variables were observed in both ionization modes, with two main dense regions of loadings (better shown in the loading plots in Figure S11), which respectively appeared correlated with the c1 and c2 sample groups.

**Figure 5:**
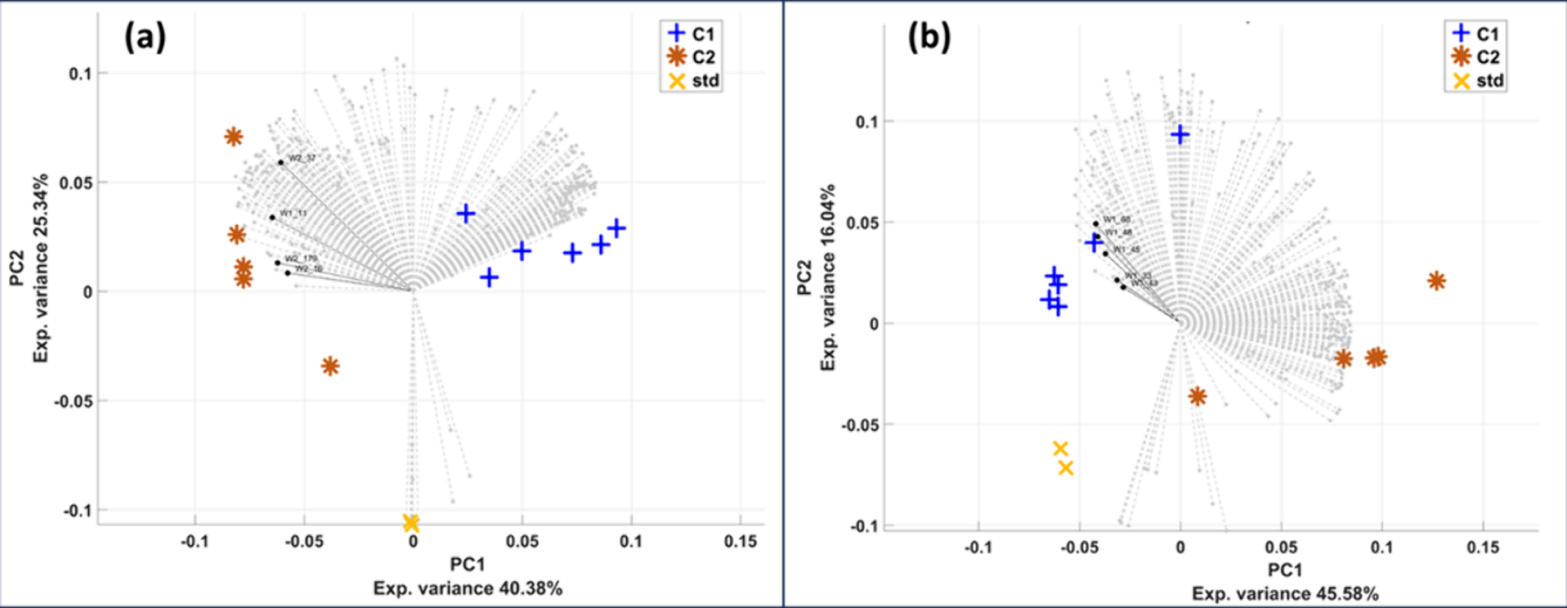
PCA biplot of MCR elution peak areas obtained in (a) positive ionization mode and (b) negative ionization mode. The sample scores are indicated using different symbols, while variable (MCR elution profile peak areas) loadings are indicated by gray circles. Loadings highlighted with black symbols were the identified MCR components that were important in distinguishing the two sample groups (see some examples explained in the text).

The following annotated MCR components were important in distinguishing the two sample groups in the positive ionization mode: paracetamol (an anti-inflammatory drug), triethyl citrate (a personal care product found in deodorants), berberine (a hypolipidemic natural substance), and *N*-oleoyl-phenylalanine (a surfactant), indicated as W1_11, W2_16, W2_37, and W2_179 in Figure 5a. These MCR components had higher peak areas in c2 samples.

As for the data analyzed in negative ionization mode, several annotated phenolic glycosides were relevant for the distinction between sample groups, namely, salicylic acid β-d-glucoside, eugenyl glucoside, ethylvanillin glucoside, sphalleroside A, and (*R*)-apiumetin glucoside (components W1_33, W1_43, W1_45, W1_48, and W1_68 in Figure 5b). The associated MCR components were characterized by higher elution peak areas in c1 samples.

A more detailed discussion on the group discrimination highlighted by PCA is given in Supporting Information (section S5). Other MCR components resulted significant in discriminating between c1 and c2 samples, but they could not be annotated yet, thus requiring additional investigation.

## Conclusions

The described case study allowed a systematic definition of a metric system for the evaluation of chemical reliability of the MCR components derived from the application of ROIMCR to non-target LC-HRMS/MS DIA data.

After careful evaluation of the obtained models, the combined ROIMCR-MSident workflow allowed the resolution and tentative identification of approximately 100 compounds in extracts coming from POCIS deployed in Antarctica. These results represent a step forward in the use of the ROIMCR-MSident methodology, directly linking MS1 and MS2 signals of many MCR resolved components and significantly reducing the time required for their tentative annotation.

The systematic classification of ROIMCR components at different quality levels complements their evaluation, not only based on MCR model fitting parameters (*R* ^2^ and Lof) but also on the characteristics of their MCR resolved elution and spectral profiles. In this context, a future automatization of the classification process is desirable, using the proposed quality levels and classes as a metric.

## Supplementary Material





## Notes

Details on chemicals, samples, and instrumental analysis; examples of chromatograms; additional details on the ROIMCR models; elution and spectral profiles of examples of MCR components; details on the classification system including class examples; class distribution of all ROIMCR models; details on the PCA (PDF)
List of annotated compounds for positive and negative ionizations (XLSX)

## References

[1] Perez
de Souza L., Alseekh S., Scossa F., Fernie A. R. (2021). Ultra-High-Performance
Liquid Chromatography High-Resolution Mass Spectrometry Variants for
Metabolomics Research. Nat. Methods.

[2] Paszkiewicz M., Godlewska K., Lis H., Caban M., Białk-Bielińska A., Stepnowski P. (2022). Advances in Suspect Screening and Non-Target Analysis
of Polar Emerging Contaminants in the Environmental Monitoring. TrAC, Trends Anal. Chem..

[3] Kutlucinar K. G., Hann S. (2021). Comparison of Preconcentration Methods
for Nontargeted Analysis of
Natural Waters Using HPLC-HRMS: Large Volume Injection versus Solid-Phase
Extraction. ELECTROPHORESIS.

[4] Hollender J., Schymanski E. L., Singer H. P., Ferguson P. L. (2017). Nontarget Screening
with High Resolution Mass Spectrometry in the Environment: Ready to
Go?. Environ. Sci. Technol..

[5] González-Gaya B., Lopez-Herguedas N., Bilbao D., Mijangos L., Iker A. M., Etxebarria N., Irazola M., Prieto A., Olivares M., Zuloaga O. (2021). Suspect and
Non-Target Screening: The Last Frontier
in Environmental Analysis. Anal. Methods.

[6] Köppe T., Jewell K. S., Dietrich C., Wick A., Ternes T. A. (2020). Application
of a Non-Target Workflow for the Identification of Specific Contaminants
Using the Example of the Nidda River Basin. Water Res..

[7] Buzier R., Guibal R., Lissalde S., Guibaud G. (2019). Limitation of Flow
Effect on Passive Sampling Accuracy Using POCIS with the PRC Approach
or O-DGT: A Pilot-Scale Evaluation for Pharmaceutical Compounds. Chemosphere.

[8] Djomte V. T., Taylor R. B., Chen S., Booij K., Chambliss C. K. (2018). Effects
of Hydrodynamic Conditions and Temperature on Polar Organic Chemical
Integrative Sampling Rates. Environ. Toxicol.
Chem..

[9] Benedetti B., Baglietto M., MacKeown H., Scapuzzi C., Di Carro M., Magi E. (2022). An Optimized Processing Method for Polar Organic Chemical Integrative
Samplers Deployed in Seawater: Toward a Maximization of the Analysis
Accuracy for Trace Emerging Contaminants. J.
Chromatogr. A.

[10] Sunyer-Caldú A., Benedetti B., Valhondo C., Martínez-Landa L., Carrera J., Di Carro M., Magi E., Diaz-Cruz M. S. (2023). Using Integrative
Samplers to Estimate the Removal of Pharmaceuticals and Personal Care
Products in a WWTP and by Soil Aquifer Treatment Enhanced with a Reactive
Barrier. Sci. Total Environ..

[11] MacKeown H., Benedetti B., Di Carro M., Magi E. (2022). The Study of Polar
Emerging Contaminants in Seawater by Passive Sampling: A Review. Chemosphere.

[12] Alygizakis N., Giannakopoulos T., Τhomaidis N. S., Slobodnik J. (2022). Detecting
the Sources of Chemicals in the Black Sea Using Non-Target Screening
and Deep Learning Convolutional Neural Networks. Sci. Total Environ..

[13] Bonnefille B., Karlsson O., Rian M. B., Raqib R., Parvez F., Papazian S., Islam M. S., Martin J. W. (2023). Nontarget Analysis
of Polluted Surface Waters in Bangladesh Using Open Science Workflows. Environ. Sci. Technol..

[14] Eysseric E., Beaudry F., Gagnon C., Segura P. A. (2021). Non-Targeted Screening
of Trace Organic Contaminants in Surface Waters by a Multi-Tool Approach
Based on Combinatorial Analysis of Tandem Mass Spectra and Open Access
Databases. Talanta.

[15] Helmus R., Ter Laak T. L., Van Wezel A. P., De Voogt P., Schymanski E. L. (2021). patRoon:
Open Source Software Platform for Environmental Mass Spectrometry
Based Non-Target Screening. J. Cheminform.

[16] González-Gaya B., Lopez-Herguedas N., Bilbao D., Mijangos L., Iker A. M., Etxebarria N., Irazola M., Prieto A., Olivares M., Zuloaga O. (2021). Suspect and Non-Target Screening: The Last Frontier
in Environmental Analysis. Anal. Methods.

[17] Gago-Ferrero P., Schymanski E. L., Bletsou A. A., Aalizadeh R., Hollender J., Thomaidis N. S. (2015). Extended Suspect and Non-Target Strategies
to Characterize Emerging Polar Organic Contaminants in Raw Wastewater
with LC-HRMS/MS. Environ. Sci. Technol..

[18] Trostel L., Coll C., Fenner K., Hafner J. (2023). Combining Predictive
and Analytical Methods to Elucidate Pharmaceutical Biotransformation
in Activated Sludge. Environ. Sci.: Processes
Impacts.

[19] Zhao J.-H., Hu L.-X., Xiao S., Zhao J.-L., Liu Y.-S., Yang B., Zhang Q.-Q., Ying G.-G. (2023). Screening and Prioritization
of Organic Chemicals in a Large River Basin by Suspect and Non-Target
Analysis. Environ. Pollut..

[20] Tisler S., Engler N., Jørgensen M. B., Kilpinen K., Tomasi G., Christensen J. H. (2022). From Data
to Reliable Conclusions: Identification and
Comparison of Persistent Micropollutants and Transformation Products
in 37 Wastewater Samples by Non-Target Screening Prioritization. Water Res..

[21] Solliec M., Roy-Lachapelle A., Storck V., Callender K., Greer C. W., Barbeau B. (2021). A Data-Independent Acquisition Approach
Based on HRMS to Explore the Biodegradation Process of Organic Micropollutants
Involved in a Biological Ion-Exchange Drinking Water Filter. Chemosphere.

[22] Tsugawa H., Cajka T., Kind T., Ma Y., Higgins B., Ikeda K., Kanazawa M., VanderGheynst J., Fiehn O., Arita M. (2015). MS-DIAL: Data-Independent MS/MS Deconvolution
for Comprehensive Metabolome Analysis. Nat.
Methods.

[23] Lassen J., Nielsen K. L., Johannsen M., Villesen P. (2021). Assessment of XCMS
Optimization Methods with Machine-Learning Performance. Anal. Chem..

[24] Gorrochategui E., Jaumot J., Tauler R. (2019). ROIMCR: A
Powerful Analysis Strategy
for LC-MS Metabolomic Datasets. BMC Bioinformatics.

[25] Pérez-López C., Oró-Nolla B., Lacorte S., Tauler R. (2023). Regions of Interest
Multivariate Curve Resolution Liquid Chromatography with Data-Independent
Acquisition Tandem Mass Spectrometry. Anal.
Chem..

[26] MacKeown H., Scapuzzi C., Baglietto M., Benedetti B., Di Carro M., Magi E. (2024). Wastewater and Seawater
Monitoring
in Antarctica: Passive Sampling as a Powerful Strategy to Evaluate
Emerging Pollution. Sci. Total Environ..

[27] Tautenhahn R., Böttcher C., Neumann S. (2008). Highly Sensitive Feature Detection
for High Resolution LC/MS. BMC Bioinformatics.

[28] Pérez-Cova M., Bedia C., Stoll D. R., Tauler R., Jaumot J. (2021). MSroi: A Pre-Processing
Tool for Mass Spectrometry-Based Studies. Chemom.
Intell. Lab. Syst..

[29] De
Juan A., Jaumot J., Tauler R. (2014). Multivariate Curve Resolution (MCR).
Solving the Mixture Analysis Problem. Anal.
Methods.

[30] Jaumot J., de Juan A., Tauler R. (2015). MCR-ALS GUI 2.0: New Features and
Applications. Chemom. Intell. Lab. Syst..

[31] Dalmau N., Bedia C., Tauler R. (2018). Validation
of the Regions of Interest
Multivariate Curve Resolution (ROIMCR) Procedure for Untargeted LC-MS
Lipidomic Analysis. Anal. Chim. Acta.

[32] Perez-Lopez C., Ginebreda A., Carrascal M., Barcelò D., Abian J., Tauler R. (2021). Non-Target
Protein Analysis of Samples
from Wastewater Treatment Plants Using the Regions of Interest-Multivariate
Curve Resolution (ROIMCR) Chemometrics Method. J. Environ. Chem. Eng..

[33] Tauler R., Gorrochategui E., Jaumot J. (2015). A Protocol for LC-MS
Metabolomic
Data Processing Using Chemometric Tools. Protocols
Exchange.

[34] Marín-García M., De Luca M., Ragno G., Tauler R. (2022). Coupling of Spectrometric,
Chromatographic, and Chemometric Analysis in the Investigation of
the Photodegradation of Sulfamethoxazole. Talanta.

[35] Windig W., Guilment J. (1991). Interactive Self-Modeling
Mixture Analysis. Anal. Chem..

[36] Gorrochategui E., Casas J., Porte C., Lacorte S., Tauler R. (2015). Chemometric
Strategy for Untargeted Lipidomics: Biomarker Detection and Identification
in Stressed Human Placental Cells. Anal. Chim.
Acta.

[37] Perez-Lopez C., Ginebreda A., Jaumot J., Yamamoto F. Y., Barcelo D., Tauler R. (2024). MSident: Straightforward Identification of Chemical
Compounds from MS-Resolved Spectra. Chemom.
Intell. Lab. Syst..

[38] Vrana B., Allan I. J., Greenwood R., Mills G. A., Dominiak E., Svensson K., Knutsson J., Morrison G. (2005). Passive Sampling Techniques
for Monitoring Pollutants in Water. TrAC Trends
Anal. Chem..

[39] Cerrato A., Bedia C., Capriotti A. L., Cavaliere C., Gentile V., Maggi M., Montone C. M., Piovesana S., Sciarra A., Tauler R., Laganà A. (2021). Untargeted
Metabolomics of Prostate Cancer Zwitterionic and Positively Charged
Compounds in Urine. Anal. Chim. Acta.

[40] Lotfi
Khatoonabadi R., Vosough M., Hohrenk L. L., Schmidt T. C. (2021). Employing
Complementary Multivariate Methods for a Designed Nontarget LC-HRMS
Screening of a Wastewater-Influenced River. Microchem. J..

[41] Majors R. E. (2003). The Cleaning
and Regeneration of Reversed Phase HPLC Columns. LGGC North America.

[42] Schymanski E. L., Jeon J., Gulde R., Fenner K., Ruff M., Singer H. P., Hollender J. (2014). Identifying Small Molecules via High
Resolution Mass Spectrometry: Communicating Confidence. Environ. Sci. Technol..

[44] Ahmadi S., Winter D. (2018). Identification of Poly­(Ethylene
Glycol) and Poly­(Ethylene
Glycol)-Based Detergents Using Peptide Search Engines. Anal. Chem..

[100] Navarro-Reig M., Jaumot J., Tauler R. (2018). An untargeted
lipidomic
strategy combining comprehensive two-dimensional liquid chromatography
and chemometric analysis. J. Chromatogr. A.

[45] Yamamoto F. Y., Pérez-López C., Lopez-Antia A., Lacorte S., de Souza Abessa D. M., Tauler R. (2023). Linking MS1 and MS2
Signals in Positive and Negative Modes of LC-HRMS in Untargeted Metabolomics
Using the ROIMCR Approach. Anal. Bioanal. Chem..

