# Supplementary material for: Improving Regions of Interest Multivariate Curve Resolution: Development of an Empirical Metric System through the Study of Passive Sampling Extracts of Wastewater in Antarctica
Source: Anal Chem. 2025 Jun 13;97(25):13110–9. doi: 10.1021/acs.analchem.5c00777 (PMC12224161; doi:10.1021/acs.analchem.5c00777)
Supplement: Supplementary file 1 [file ac5c00777_si_001.pdf]

Supporting Information

Improving Regions Of Interest Multivariate Curve Resolution: development of an empirical metric system through the study of passive sampling extracts of wastewater in Antarctica

Barbara Benedetti[1], Carlos Perez-Lopez[2], Henry MacKeown[1], Emanuele Magi[1], Roma Tauler[2]

[1] *Department of Chemistry and Industrial Chemistry, University of Genoa, Genoa, Italy*

[2] *Institute of Environmental Assessment and Water Studies (IDAEA-CSIC), Department of Environmental Chemistry, Barcelona, Spain*

Table of Contents

Section S1: Chemicals

Table S1. Details on the Antarctic samples analyzed in the study: sampling campaign, POCIS deployment period and samples name. Six samples came from sample campaign 1 (c1) and 5 samples from sample campaign 2 (c2).

Section S2: Details on the UHPLC-MS/MS Instrumental analysis

Table S2. Gradient elution used in the LC-HRMS analysis in both ESI positive acquisition and ESI negative acquisition runs.

Figure S1: chromatogram obtained for sample C1P1 after MS1 ROI processing (positive acquisition data). The profiles of the extracted ROIs (in different colors) are overlapped and the time windowing is highlighted as follows: (a)time window W1; (b)time window W2; (c)time window W3.

Figure S2: chromatogram obtained for sample C1P1 after MS1 ROI processing (negative acquisition data). The profiles of the extracted ROIs (in different colors) are overlapped and the time windowing is highlighted as follows: (a)time window W1; (b)time window W2; (c)time window W3.

Table S3: Number of m/z values (ROI) obtained for the individual data sets (campaign 1 and campaign 2, separately) and number of ROIs obtained after column-wise matrix augmentation (campaign 1 + campaign 2), for the two ionization modes.

Section S3: Preliminary MCR-ALS modelling tests

Table S4: MCR fitting results and number of components of all tested models on the different time windows. The symbols “(+)” and “(-)” indicate positive and negative acquisition modes, respectively.

Figure S3. elution (a) and spectral (b) profiles of the MCR component 2 from time window data section W1 (positive ionization data), annotated as styrene; elution (c) and spectral (d) profiles of the MCR component 37 from time window data section W2 (positive ionization data), annotated as berberine. The elution profiles include the chromatographic profile of all the samples, displayed one next to the other. A clear, single peak with good shape indicates a satisfactory component.

Figure S4. elution (a) and spectral (b) profiles of the MCR component 26 from time window data section W1 (negative ionization data), annotated as O-Demethylfonsecin; elution (c) and spectral (d) profiles of the MCR component 47 from time window data section W1 (negative ionization data), annotated as Didesethylflurazepam.

Section S4: Detailed description of the classification system

Table S5: Possible peaks in the MS1 spectrum in positive ionization mode. Most common adducts and more abundant isotopic peaks are considered.

Table S6: Possible peaks in the MS1 spectrum in negative ionization mode. Most common adducts and more abundant isotopic peaks are considered.

Figure S5: Detailed examples of MCR components classified as B1 (a); B2 (b); B3(c); and B4 (d). Elution and spectra profiles were characterized by different quality levels, as explained in Table 2. These MCR components were not annotated.

Figure S6: Detailed examples of MCR components belonging to class: C1 (a); C2 (b). Both Elution and spectral profiles are characterized by rather low quality or "anomalies" are observed (see description in Table 3).

Figure S7: Class distribution of the resolved MCR components in all the computed models. The signs "(+)" and "(-)" indicate the polarity of the acquisition mode, while W1, W2 and W3 indicate the time window of the chromatograms (see Materials and Method section for details).

Figure S8. Spectral profile of component 89 from time window W3 of the data obtained by positive ionization mode. The peaks in MS1, equally spaced (difference of 44 Da), probably indicate a polymeric chemical, characterized by in-source fragmentation.

Section S5: Detailed description of PCA results

Figure S9: 3D score plot on the first three principal components for the data obtained by positive ionization mode.

Figure S10:3D score plot on the first three principal components for the data obtained by negative ionization mode.

Figure S11: Loadings plots on the first two principal components for the data obtained by positive ionization mode(a) and negative ionization mode (b). The input for the PCA was the matrix including the peak area of all resolved MCR components of the models for all samples and standards.

S1. Chemicals

Analytical standards were purchased from different suppliers: acesulfame K (ACS), atenolol (ATN), benzophenone-3 (BP-3), bisphenol A (BPA), carbamazepine (CBZ), clenbuterol (CLBT), chloramphenicol (CMPH), chlormequat (CMQ), cocaine (COCA), daminozide (DMNZ), 2,4-dichlorophenoxyacetic acid (2,4-D), β-estradiol (E2), estrone (E1), 17α-ethinyl estradiol (EE2), ethyl hexyl methoxy cinnamate (EHMC), ethyl hexyl salicylate (EHS), fluroxypyr (FXP), furosemide (FRSM), gemfibrozil (GEM), hydrochlorothiazide (HCTZ), ibuprofen (IBU), mepiquat (MPQ), metformin (MTF), metoprolol (MTPL), nicotine (NCT), octocrylene (OC), octyl dimethyl p-aminobenzoate (OD-PABA), omethoate (OMT), paraxanthine (PRX), perfluorooctane sulfonate (PFOS), perfluorooctanoic acid (PFOA), sucralose (SCL), taurine (TRN), terbutaline (TRBT), theobromine (TBR), theophylline (TFL), and triclosan (TCS) were from Sigma-Aldrich (St. Louis, MO, USA); caffeine (CAFF), diclofenac (DCF), ketoprofen (KET) and naproxen (NAPR), from Fluka Analytical (Saint Gallen, Switzerland), while salbutamol (SLBT) from Alfa Aesar (Haverhill, MA, USA). All analytical standards were equal or above 98 % of purity. Single stock standard solutions of the analytes were prepared dissolving pure standards in methanol (MeOH) or MeOH:water, 1:1 or 1:3 (v/v), depending on their polarity, and were stored at -18 ◦C. A standard mix of the analytes was prepared at a concentration of 50 µg L^{-1} and injected along with the samples during the positive and negative ionization mode batches.

Table S 1. *Details on the Antarctic samples analyzed in the study: sampling campaign, POCIS deployment period and samples name. Six samples came from sample campaign 1 (c1) and 5 samples from sample campaign 2 (c2).*

**Antarctic sampling campaign**	**Passive sampling period (outlet of the wastewater treatment facility)**	**Sample name**
2021-2022 campaign (c1)	November 7th – 22nd 2021	POCIS C1P1
	November 22nd - December 6th 2021	POCIS C1P2
	December 6th - 21st 2021	POCIS C1P3
	December 21st - January 4th 2022	POCIS C1P4
	January 4th - 19th 2022	POCIS C1P5
	January 19th – February 2nd 2022	POCIS C1P6
2022-2023 campaign (c2)	November 3rd – 17th 2022	POCIS C2P1
	November 17th – 30th 2022	POCIS C2P2
	December 8th - 24th 2022	POCIS C2P3
	December 26th 2022 - January 11th 2023	POCIS C2P4
	January 12th - 28th 2023	POCIS C2P5

S2. Details on the UHPLC-MS/MS Instrumental analysis

The UHPLC was equipped with a binary pump, a degasser, a thermostated autosampler (maintained at 10°C) and a thermostated column compartment. The analysis temperature was 40°C and the gradient used with the pentafluorophenyl column is reported in Table S2. The UHPLC was coupled to the Q-TOF mass spectrometer through a Heated Electrospray Ionization Source (HESI). The HESI settings were: capillary voltage of 2500 V, end plate offset of 500 V, drying gas at 8 L min^{-1} and 200°C, probe gas at 4 L min^{-1} and 300°C.

Table S2. *Gradient elution used in the LC-HRMS analysis in both ESI positive acquisition and ESI negative acquisition runs.*

TIME (min)	A (H_2O)* %	B (ACN)* %
0	95	5
5	95	5
20	50	50
22	30	70
30	10	90
35	10	90
37	95	5
+ Post time 9 min		

*in the positive acquisition mode, a 0.1% of formic acid was added to the mobile phases.

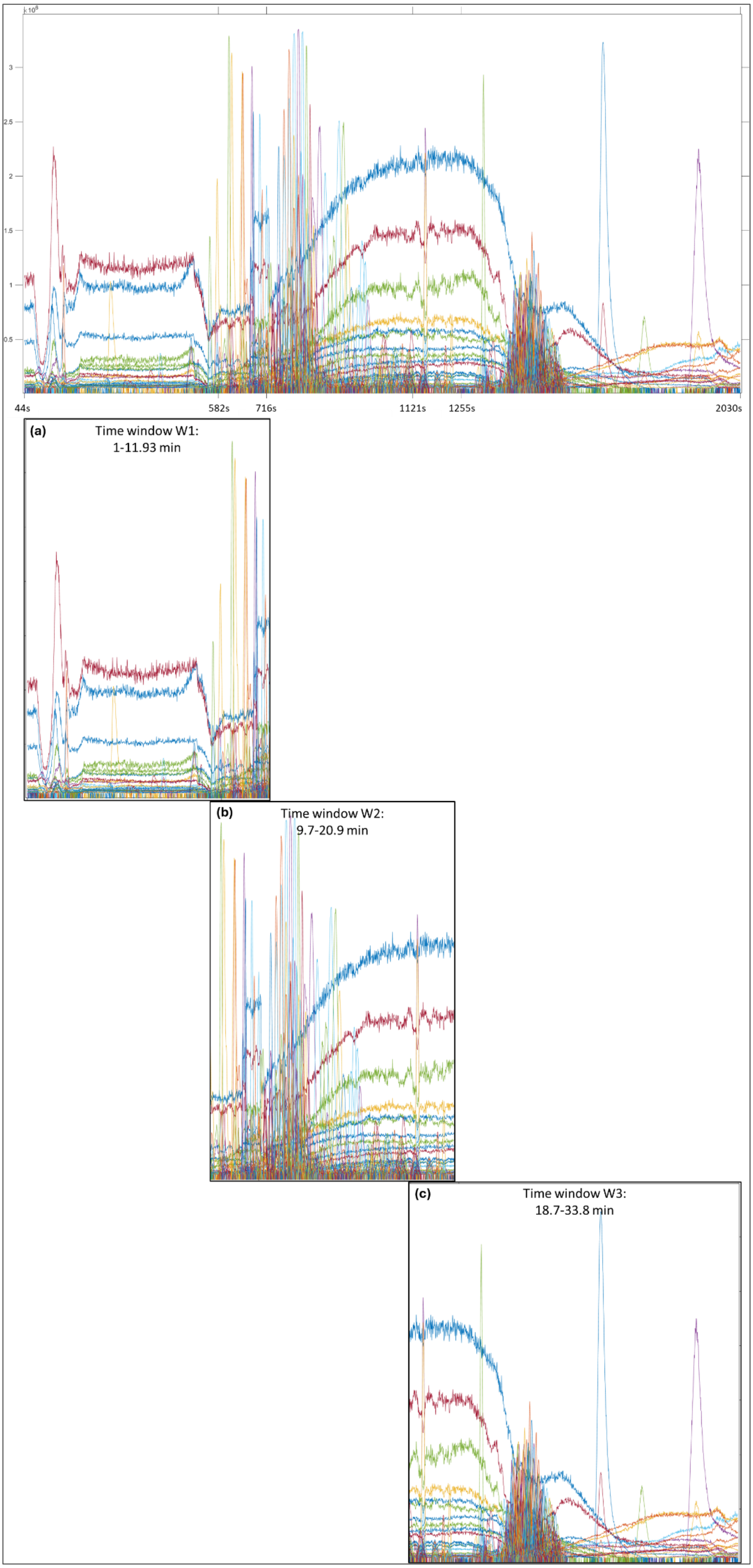

Figure S1: *chromatogram obtained for sample C1P1 after MS1 ROI processing (positive acquisition data). The profiles of the extracted ROIs (in different colors) are overlapped and the time windowing is highlighted as follows: (a)time window W1; (b)time window W2; (c)time window W3.*

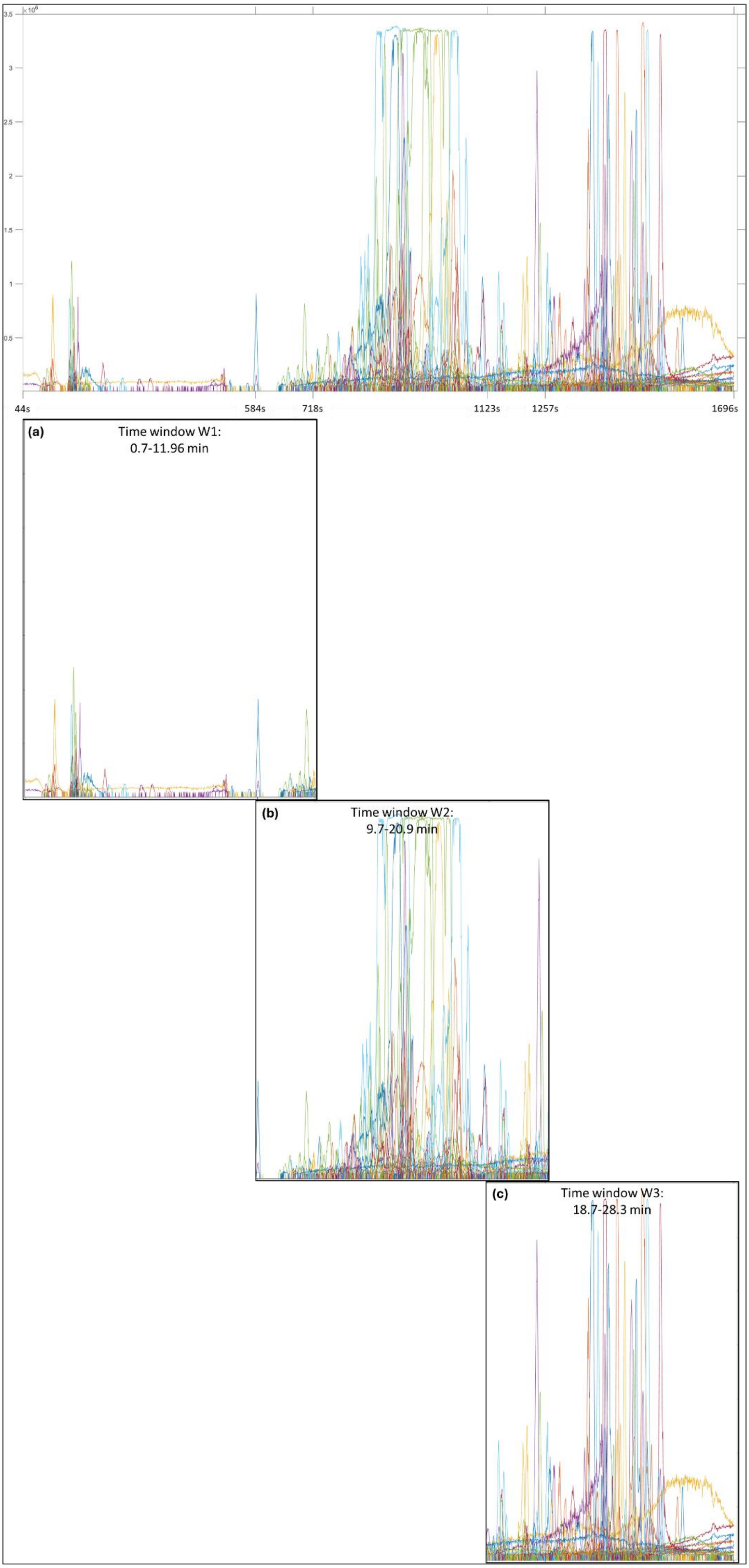

Figure S2: *chromatogram obtained for sample C1P1 after MS1 ROI processing (negative acquisition data). The profiles of the extracted ROIs (in different colors) are overlapped and the time windowing is highlighted as follows: (a)time window W1; (b)time window W2; (c)time window W3.*

Table S3: *Number of m/z values (ROI) obtained for the individual data sets (campaign 1 and campaign 2, separately) and number of ROIs obtained after column-wise matrix augmentation (campaign 1 + campaign 2), for the two ionization modes.*

**SAMPLE SET**	**MSroi matrix**	**ROI number (after blank subtraction)**	**Total number of ROIs (MS1+MS2 row-wise augmentation)**
Campaign 1, positive ionization mode (6 samples)	c1(+)**MS1**roi	2586	4896
	c1(+)**MS2**roi	2310	
Campaign 2, positive ionization mode (5 samples)	c2(+)**MS1**roi	2176	4699
	c2(+)**MS2**roi	2523	
Campaign 1+2 (merged)*, positive ionization mode (11 samples)	c1-c2(+)**MS1**roi	3169	**6307**
	c1-c2(+)**MS2**roi	3138	
Campaign 1, negative ionization mode (6 samples)	c1(-)**MS1**roi	801	2439
	c1(-)**MS2**roi	1638	
Campaign 2, negative ionization mode (5 samples)	c2(-)**MS1**roi	1066	3229
	c2(-)**MS2**roi	2163	
Campaign 1+2 (merged)*, negative ionization mode (11 samples)	c1-c2(-)**MS1**roi	1293	**3863**
	c1-c2(-)**MS2**roi	2570	

*The data matrices of campaign 1 and 2 were merged by a column-wise matrix augmentation, for each ionization mode, respectively.

S3. Preliminary MCR-ALS modelling tests

The complexity of the data set made it necessary to proceed with the computation of different MCR models, to finally achieve the most appropriate one. At first, the whole chromatographic profile of the data acquired in positive ionization mode was considered for preliminary evaluations. Starting from the total obtained ROIs and the Singular Value Decomposition, a number of components ranging from 90 to 210 was set for the MCR modelling, with explained variances and lack of fit (lof) values passing from 88 to 95% and from 33% to 21%, respectively. The construction of these models was quite laborious in terms of computation time, and the lof values were considered not satisfactory. Moreover, a low number of tentative identifications were reached through the use of MSident. For this reason, time windowing of the chromatograms was pondered, and MCR-ALS was applied by testing a division into two time windows and three time windows. The results obtained by dividing

the chromatograms into two sections gave slightly better results, suggesting that cutting the profiles to simplify the computation might be promising. Therefore, a final subdivision into 3 time windows was selected, and for each one, different number of components were tested until reaching satisfactory values of explained variances and lof (explained variances at least equal to 95% and lof ≤ 20%). These conditions were not satisfied for the third time window (positive ionization data), despite performing a definitely higher number of tests (as shown in Table S4). A noisier profile toward the end of the chromatographic run probably hindered the good fitting of the model, as described in the main text.
Given these results on the data in positive ionization mode, those acquired in negative ionization were directly processed by following the workflow involving three time windows subdivision.
Once again, different numbers of components were tested, and the explained variances and lof values obtained in each test are reported in Table S4. Only the optimal final models are described in the main manuscript.

Table S4*: MCR fitting results and number of components of all tested models on the different time windows. The symbols "(+)" and "(-)" indicate positive and negative acquisition modes, respectively.*

MCR Model	Number of MCR components	R^2 [a]	Lof [b]
Model (+) W1	40	95.6%	20.9%
Model (+) W1	50	97.2%	16.6%
Model (+) W1	***60***	***98.24%***	***13.3%***
Model (+) W2	140	95.2%	21.9%
Model (+) W2	160	95.9%	20.2%
Model (+) W2	***180***	***97.11%***	***17.0%***
Model (+) W3	60	89.8%	31.9%
Model (+) W3	100	93.4%	25.6%
Model (+) W3	120	94.5%	23.4%
Model (+) W3	***140***	***95.02%***	***22.3%***
Model (+) W3	160	94.5%	23.5%
Model (+) W3	180	94.3%	23.8%
Model (-) W1	60	95.9%	20.1%
Model (-) W1	80	97.3%	16.6%
Model (-) W1	***100***	***97.96%***	***14.3%***
Model (-) W2	150	97.3%	16.5%
Model (-) W2	160	97.7%	15.1%
Model (-) W2	***190***	***98.22%***	***13.4%***
Model (-) W3	140	98.4%	12.7%
Model (-) W3	120	98.4%	12.6%
Model (-) W3	***100***	***98.63%***	***11.7 %***

a- % of variance explained by the model
b- % of lack of fit: parameter related to the residuals not explained by the MCR model (the lower the better)

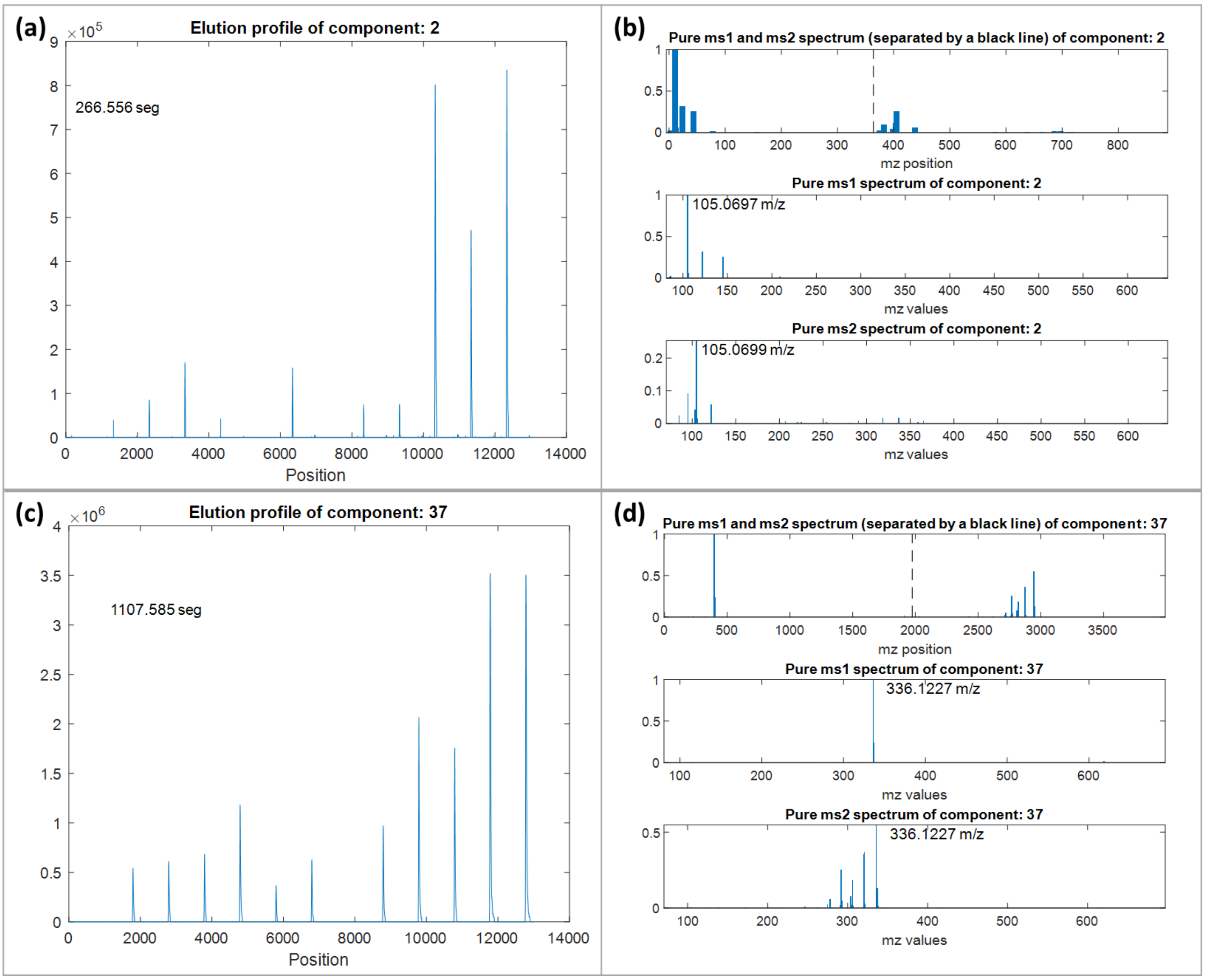

Figure S3. *elution (a) and spectral (b) profiles of the MCR component 2 from time window data section W1 (positive ionization data), annotated as styrene; elution (c) and spectral (d) profiles of the MCR component 37 from time window data section W2 (positive ionization data), annotated as berberine. The elution profiles include the chromatographic profile of all the samples, displayed one next to the other. A clear, single peak with good shape indicates a satisfactory component.*

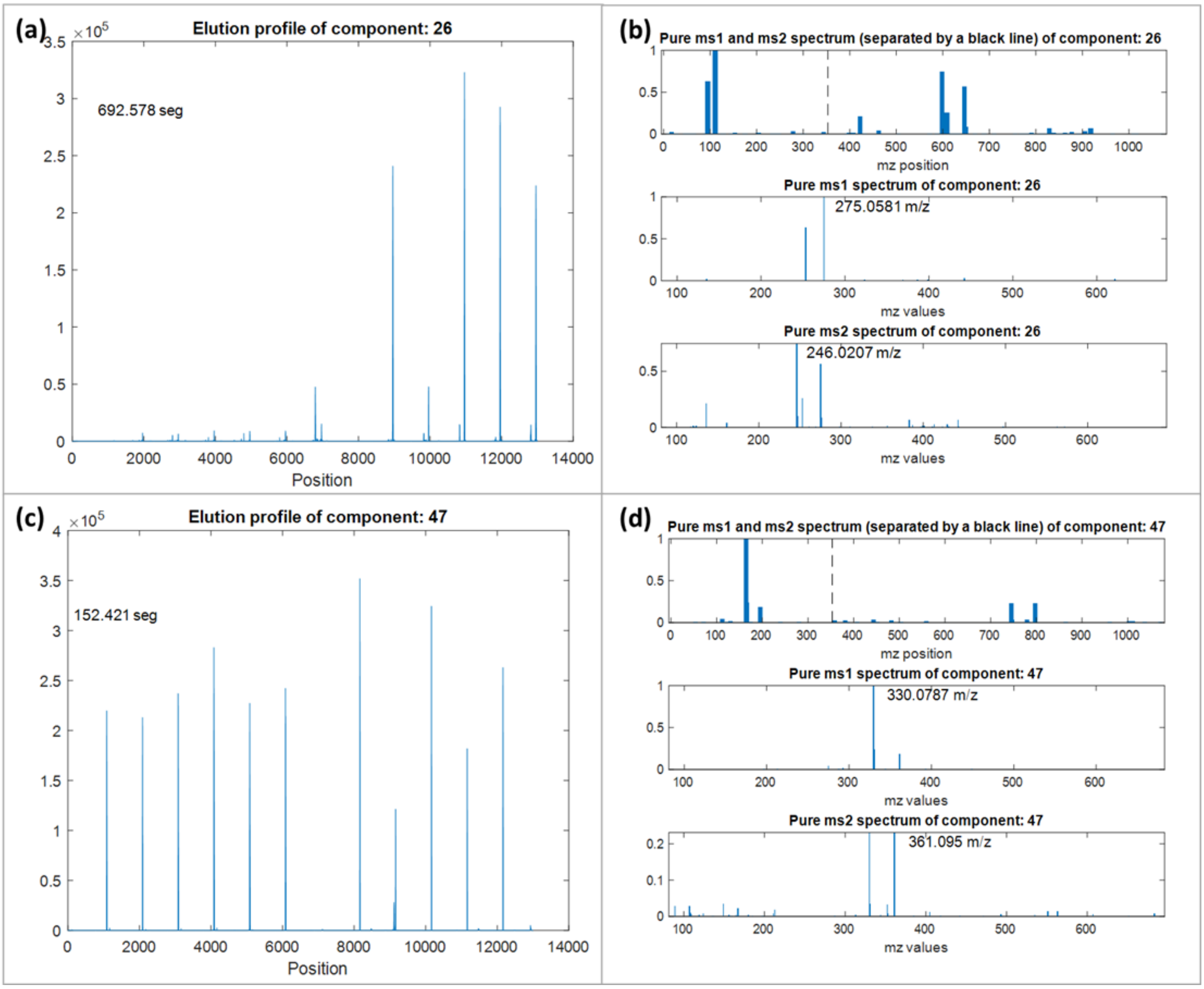

Figure S4*. elution (a) and spectral (b) profiles of the MCR component 26 from time window data section W1 (negative ionization data), annotated as O-Demethylfonsecin; elution (c) and spectral (d) profiles of the MCR component 47 from time window data section W1 (negative ionization data), annotated as Didesethylflurazepam.*

S4. Detailed description of the classification system

In order to establish the quality of the MCR components of the models obtained for the different time windows, some criteria were compiled, based on the principles of atmospheric pressure ionization-tandem mass spectrometry and liquid chromatography. In particular, these two aspects were considered:

- the shape and number of the peaks observed in the chromatographic profile;
- the number of mass peaks observed in the MS1 and MS2 spectrum, their m/z values and their relative abundance.

By visually checking the MCR components, seven main classes appeared to represent all encountered cases. They were grouped depending on a “quality” level, related to good (level A), acceptable (level B) or unacceptable (level C) characteristics of both the spectrum and chromatogram.

Class A

Class A components are probably associated to single compounds, with a spectrum that allows a possible annotation using spectral databases.

- **Spectrum: good quality**
    (1). A maximum of 3-4 main peaks on MS1 are observed, with a plausible m/z distance (considering the possible adducts in positive and negative ionization), and their corresponding isotopic peaks.

Table S5*: Possible peaks in the MS1 spectrum in positive ionization mode. Most common adducts and more abundant isotopic peaks are considered.*

	ION	m/z (no halogens)	m/z (halogens)
Precursor 1 (P1)	$(M+H)^+$	X	X
P1 isotopic peak		X+1[a]	X+2
		X+2[a]	X+4[b]
Precursor 2 (P2)	$(M+Na)^+$	X+21.9819	X+21.9819
P2 isotopic peak		X+22.98[c]	X+23.98[c]
		X+23.98[c]	X+25.98[c]
Precursor 3 (P3)	$(M+NH_4)^+$	X+17.0265	X+17.0265
P3 isotopic peak		X+18.03[c]	X+19.03[c]
		X+19.03[c]	X+21.03[c]
Precursor 4 (P4)	$(M+K)^+$	X+37.9559	X+37.9559
P4 isotopic peak		X+38.96[c]	X+39.96[c]
		X+39.96[c]	X+41.96[c]

a- The accurate m/z value of the isotopic peak may slightly differ depending on which atoms give the main contribution to the isotopic peak, thus the m/z value is rounded to X+1 or X+2. The same applies to the isotopic peaks of molecules containing halogens, where the m/z value of the isotopic peak is rounded to X+2 or X+4.

b- When more and/or different halogens are present, also the peak with m/z=X+6 is observed (e.g. for 3 Br atoms the X+6 peak has the same intensity as the X peak).

c- The m/z difference between the adduct peak and the corresponding isotopic one is rounded for the same reason described above.

Table S6*: Possible peaks in the MS1 spectrum in negative ionization mode. Most common adducts and more abundant isotopic peaks are considered.*

	ION	m/z (no halogens)	m/z (halogens)
Precursor 1 (P1)	$(M-H)^-$	X	X
P1 isotopic peak		X+1[a]	X+2
		X+2[a]	X+4[b]
Precursor 2 (P2)	$(M+Cl)^-$	X+ 35.9767	X+ 35.9767
P2 isotopic peak		X+ 36.98[c]	X+ 37.98[c]
		X+ 37.98[c]	X+ 39.98[c]
Precursor 3 (P3)	$(M+HCOO)^-$	X+46.0055	X+46.0055
P3 isotopic peak		X+47.01[c]	X+48.01[c]
		X+48.01[c]	X+50.01[c]
Precursor 4 (P4)	$(M+CH_3COO)^-$	X+60.0211	X+60.0211
P4 isotopic peak		X+61.02[c]	X+62.02[c]
		X+62.02[c]	X+64.02[c]

a- The accurate m/z value of the isotopic peak may slightly differ depending on which atoms give the main contribution to the isotopic peak, thus the m/z value is rounded to X+1 or X+2. The same applies to the isotopic peaks of molecules containing halogens, where the m/z value of the isotopic peak is rounded to X+2 or X+4.

b- When more and/or different halogens are present, also the peak with m/z=X+6 is observed (e.g. for 3 Br atoms the X+6 peak has the same intensity as the X peak).

c- The m/z difference between the adduct peak and the corresponding isotopic one is rounded for the same reason described above.

(2). Considering a precursor ion in the MS1 spectrum with a m/z value of X, the intensity of the observed X+1 peak is lower. If the X+2 and X+4 peaks are detected, no specific requirements are posed on their intensity, as their relative abundance may be rather variable depending on the molecule mass and the number and type of halogens present. Specific isotopic patterns should be visually verified.

(3). The peaks in the MS2 spectrum present lower intensities and lower or equal m/z values compared to the base peak in MS1.

- **Chromatogram: good quality**
  A single peak is observed, with gaussian shape and signal to noise ratio>10.

Class B1

Class B1 components are probably associated to single compounds, but the lack of significant product ions in the MS2 spectrum hampers the annotation.

- **Spectrum: acceptable quality**
  (1). The same requirements as for class A are fulfilled with respect to the MS1 spectrum.
  (2). The peaks in the MS2 spectrum present a relative abundance below 5% compared to the base peak in the MS1 spectrum (no detected product ions), thus probably representing noise peaks.
- **Chromatogram: good quality**
  A single peak is observed, with gaussian shape and signal to noise ratio>10.

Class B2

Class B2 components are probably associated to single compounds, but both the spectrum and chromatogram are noisy, possibly hampering the annotation.

- **Spectrum: satisfactory quality**
  (1). A maximum of 3-4 main peaks on MS1 are observed (plus possible isotopic peaks) in the MS1 spectrum, as for the A and B1 classes; other peaks with a relative abundance below 10% compared to the MS1 base peak are observed, probably associated to noise (MS1 noise peaks).
  (2). The peaks in the MS2 spectrum present lower intensities and lower or equal m/z values compared to the base peak in MS1; other peaks with a relative abundance below 10% compared to the MS2 base peak are observed (MS2 noise peaks).
- **Chromatogram: satisfactory quality**
  A single main peak is observed, with gaussian shape and signal to noise ratio>10. Secondary peaks with lower signal to noise ratio and/or not perfectly gaussian shape (noise peaks) are observed.

Class B3a

Class B3a components are probably associated to more than one compound. Still, they may represent isomers that share the same MS behavior but have different retention times, thus annotation is possible.

- **Spectrum: satisfactory quality**
  (1). A maximum of 3-4 main peaks on MS1 are observed (plus possible isotopic peaks) in the MS1 spectrum, as for the A and B1 classes; MS1 noise peaks are present.
  (2). The peaks in the MS2 spectrum present lower intensities and lower or equal m/z values than the base peak in MS1; MS2 noise peaks are present.
- **Chromatogram: not satisfactory quality**
  Two or more peaks with gaussian shape, signal to noise>10 and comparable areas are observed; noise peaks may also be present.

Class B3b

Class B3b components are probably associated to more than one compound, probably coeluting and thus not resolved by MCR. The resulting complex spectrum hinders the annotation.

- **Spectrum: not acceptable quality**
  More than 3-4 peaks (plus isotopic peaks) are observed in the MS1 spectrum and/or their m/z distance is not associable to adducts nor isotopic peaks; MS1 noise peaks are present.
- **Chromatogram: satisfactory quality**
  A single main peak is observed, with gaussian shape and signal to noise ratio>10; noise peaks may be present.

Class B4

Class B4 components are probably associated to more than one compound, not coeluting but not resolved by MCR, probably due to the presence of some common peaks in the spectral profile. The resulting complex spectrum hinders the annotation.

- **Spectrum: not acceptable quality**
  (1). More than 3-4 peaks (plus isotopic peaks) are observed in the MS1 spectrum and their m/z distance is not associable to adducts nor isotopic peaks; MS1 noise peaks may also be present.
  (2). Several MS2 peaks are present, with intensity comparable to those of some MS1 peaks (relative abundance of 80-100%) but different m/z value, suggesting the fragmentation of more than one compound.
- **Chromatogram: not satisfactory quality**
  Two or more peaks with gaussian shape, signal to noise>10 and comparable areas are observed; noise peaks may also be present.

Class C1

Class C1 components are probably not associated to any compound. The mass peaks of background or noise may be associated to a chromatographic profile by chance.

- **Spectrum: bad quality (not acceptable)**
  (1). A maximum of 3-4 main peaks on MS1 are observed (plus possible isotopic peaks) in the MS1 spectrum; MS1 noise peaks are present.
  (2). One or more peaks are observed in the MS2 spectrum with a m/z value greater than those of the MS1 peaks and/or their relative abundance are 80-100% with respect to the MS1 peaks.
- **Chromatogram: bad quality (not acceptable)**
  Very noisy chromatogram, with one or more peaks with signal to noise ratio<10 and no gaussian shape, is observed.

Class C2

Class C2 components are probably associated to noise.

- **Spectrum: bad quality (not acceptable)**
  (1). Numerous peaks of similar intensities are present in the MS1 spectrum, probably associated to noise.
  (2). Numerous peaks of similar intensities are present in the MS2 spectrum, probably associated to noise. Relative abundance of MS2 peaks up to 80-100% compared to the MS1 peaks.
- **Chromatogram: bad quality (not acceptable)**
  An aberrant and noisy chromatographic profile is observed, not associable to any peak of gaussian shape.

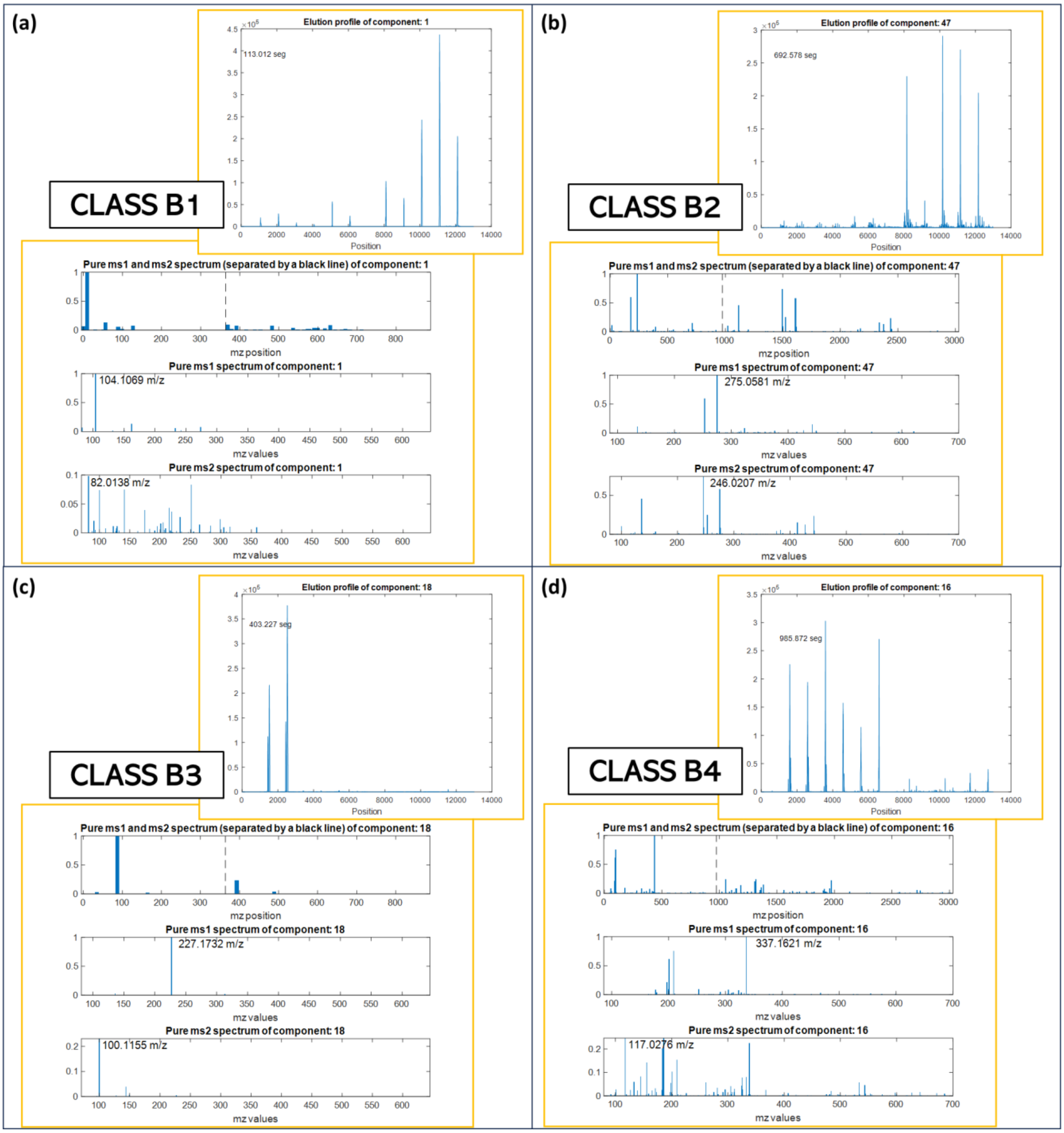

Figure S5*: Detailed examples of MCR components classified as B1 (a); B2 (b); B3(c); and B4 (d). Elution and spectra profiles were characterized by different quality levels, as explained in Table 2. These MCR components were not annotated.*

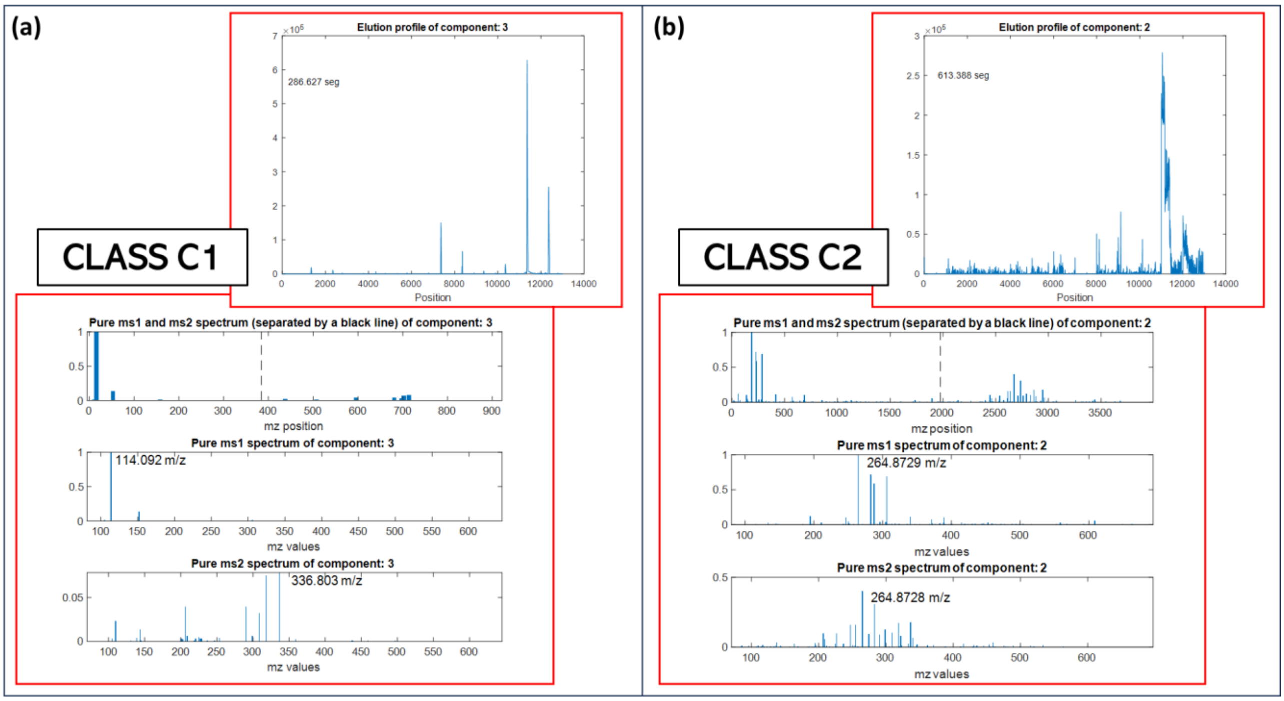

Figure S6: *Detailed examples of MCR components belonging to class: C1 (a); C2 (b). Both Elution and spectral profiles are characterized by rather low quality or "anomalies" are observed (see description in Table 3).*

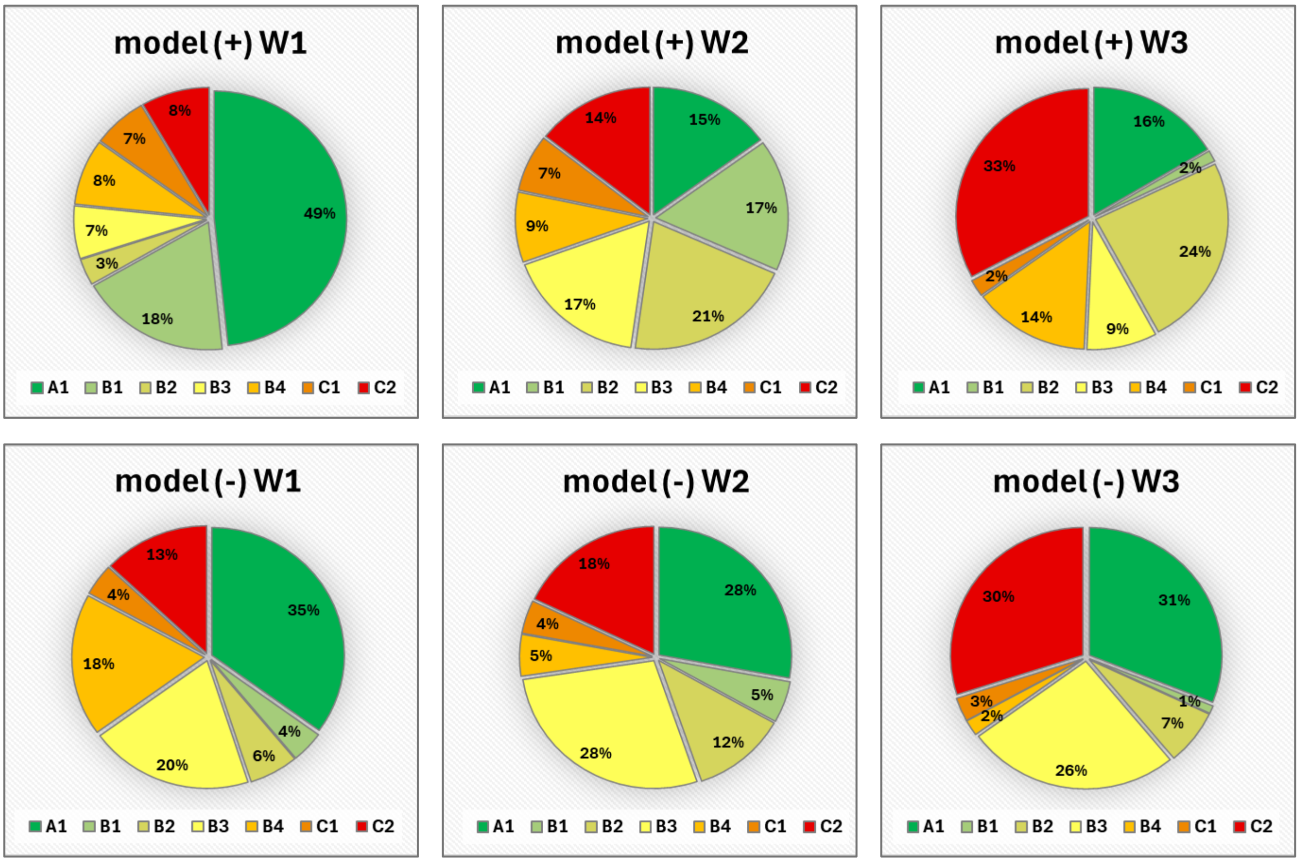

Figure S7: *Class distribution of the resolved MCR components in all the computed models. The signs "(+)" and "(-)" indicate the polarity of the acquisition mode, while W1, W2 and W3 indicate the time window of the chromatograms (see Materials and Method section for details).*

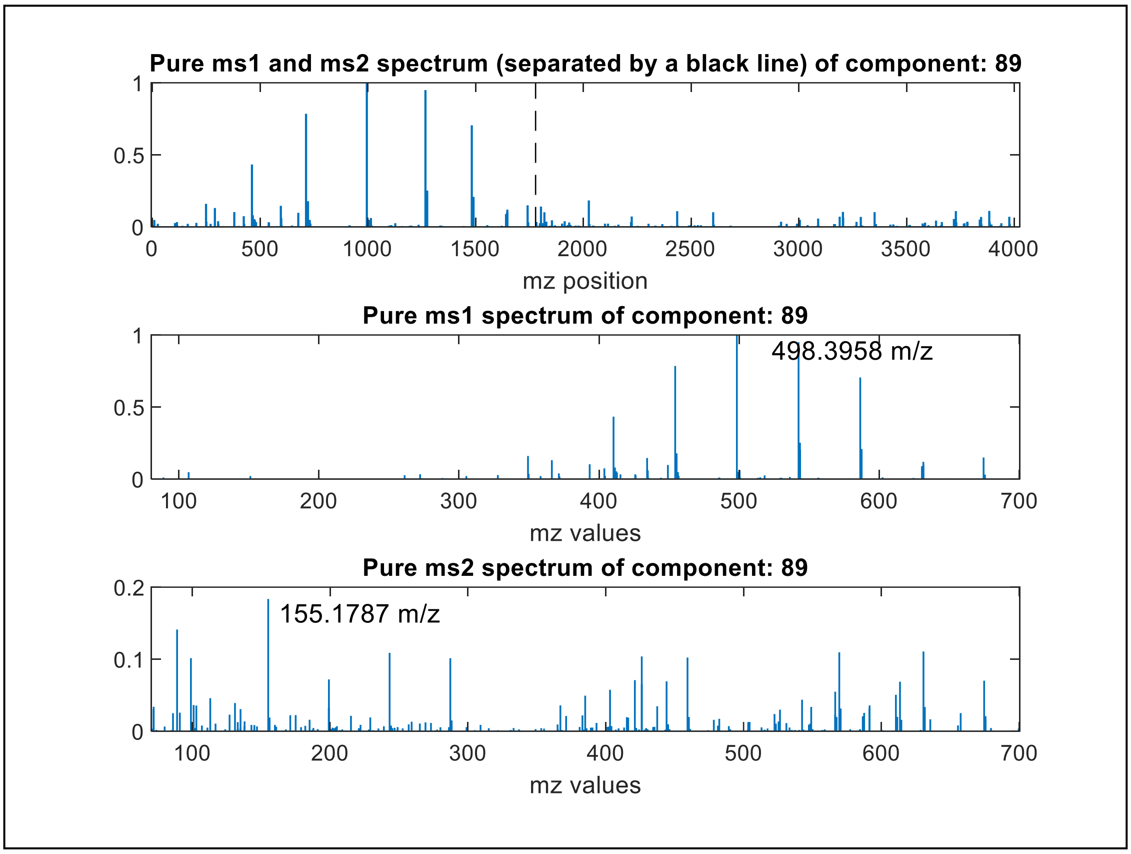

Figure S8. *Spectral profile of component 89 from time window W3 of the data obtained by positive ionization mode. The peaks in MS1, equally spaced (difference of 44 Da), probably indicate a polymeric chemical, characterized by in-source fragmentation.*

S5. Detailed description of PCA results

The 3-D scores plot in Fig. S9 and S10 highlight the separation of the sample groups in the principal components space.

Regarding the data in positive ionization mode, the main separation was along the first principal component (PC1), with c2 samples having negative scores and c1 samples having positive scores. This means that those MCR components with highly negative loadings on PC1 had higher areas in c2 samples compared to c1 samples. Among the annotated compounds, MCR component 11 from time window W1 (W1_11 in Fig. 5a), which was annotated as paracetamol, had a highly negative loading on PC1. Indeed, c2 samples presented definitely higher peak areas of this MCR component (meaning that paracetamol concentrations were higher in c2 samples than in c1 samples, in which it was even not detected in some time periods). This may be ascribed to a different availability of this pharmaceutical during the two sampling years. A similar phenomenon (but with higher concentrations in c1 samples) was observed in the targeted study for another drug (naproxen) [34]. Also, other compounds, identified as triethyl citrate (component 16 in W2, W2_16 in Fig.5a), a personal care product found in deodorants, N-Oleoyl-Phenylalanine (W2_179 in Fig.5a), a surfactant, and berberine (W2_37 in Fig. 5a), a hypolipidemic natural substance, showed negative loadings on PC1, with the respective MCR components in c2 samples having larger peak areas.

As for the data in negative ionization mode, the MCR components with negative PC1 loadings (i.e. n particular, < -0.025) and positive PC2 loadings (i.e., comprised between 0 and 0.05), were located "close" to c1 samples' scores in the biplot (Fig 5b). This indicates that the MCR components corresponding to these loadings had higher peak areas in c1 samples compared to c2 samples. Several compounds, annotated as phenolic glycosides, such as salicylic acid beta-D-glucoside (component 33

in W1, W1_33 in Fig. 5b), eugenyl glucoside (component 43 in W1, W1_43 in Fig. 5b), ethylvanillin glucoside (W1_45 in Fig. 5b), sphalleroside A (W1_48 in Fig. 5b), (R)-apiumetin glucoside (W1_68 in Fig. 5b), found in vegetables, had negative loadings on PC1 (< -0.025) and loadings below 0.05 on PC2. In fact, the associated MCR components were characterized by a larger peak area in c1 samples, maybe indicating different food availability in the two campaigns. Another interesting compound whose component showed higher peak areas in the c1 samples was the mycotoxin nivalenol; the reasons for this difference remain unknown.

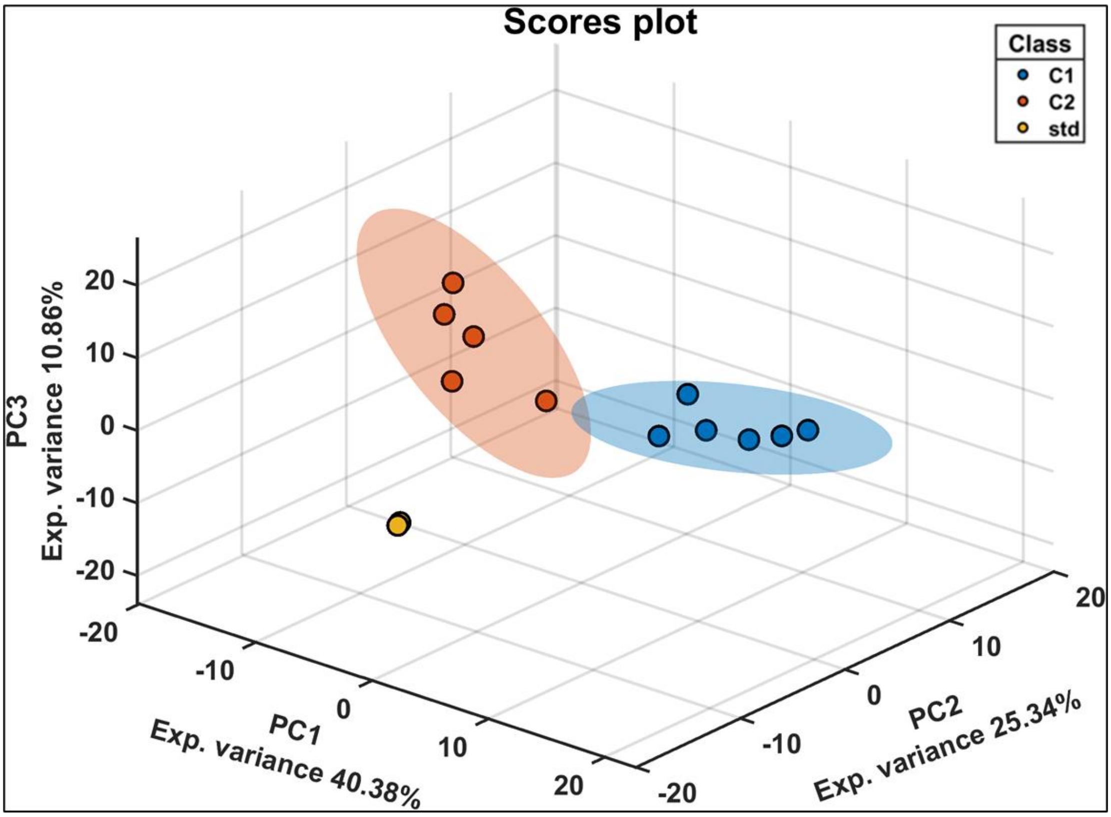

Figure S9: *3D score plot on the first three principal components for the data obtained by positive ionization mode.*

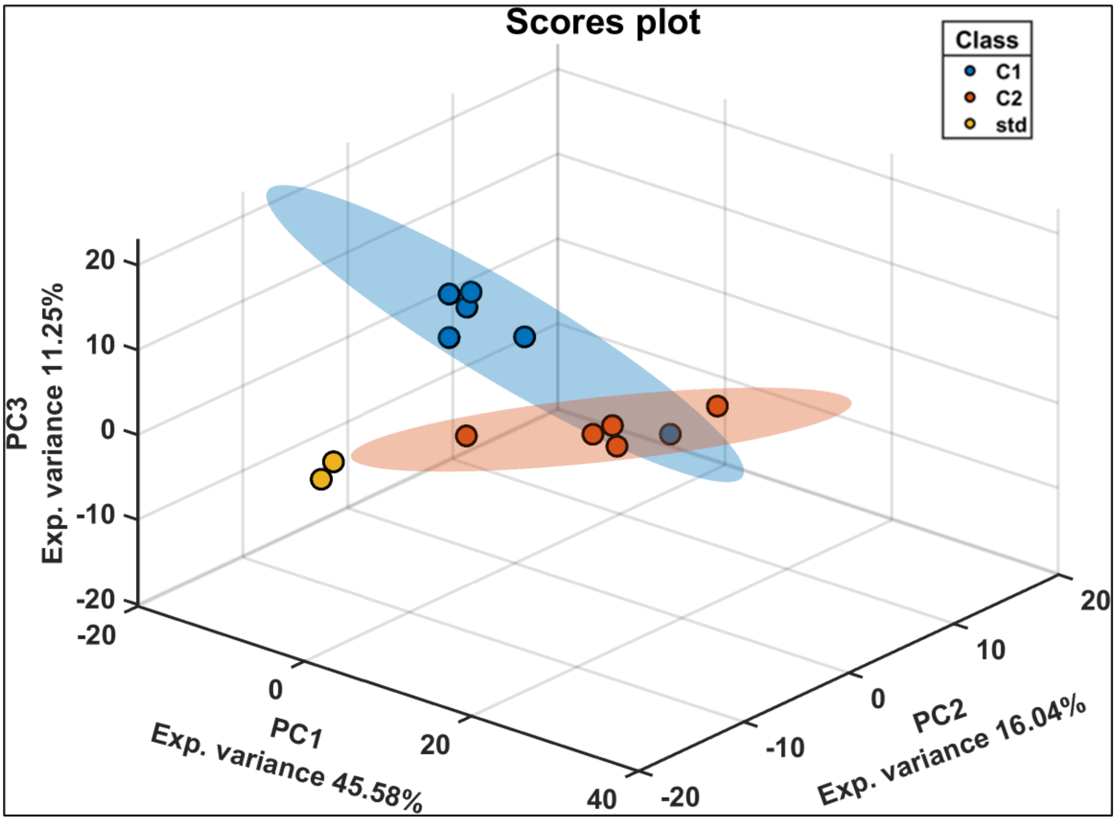

Figure S10:*3D score plot on the first three principal components for the data obtained by negative ionization mode*

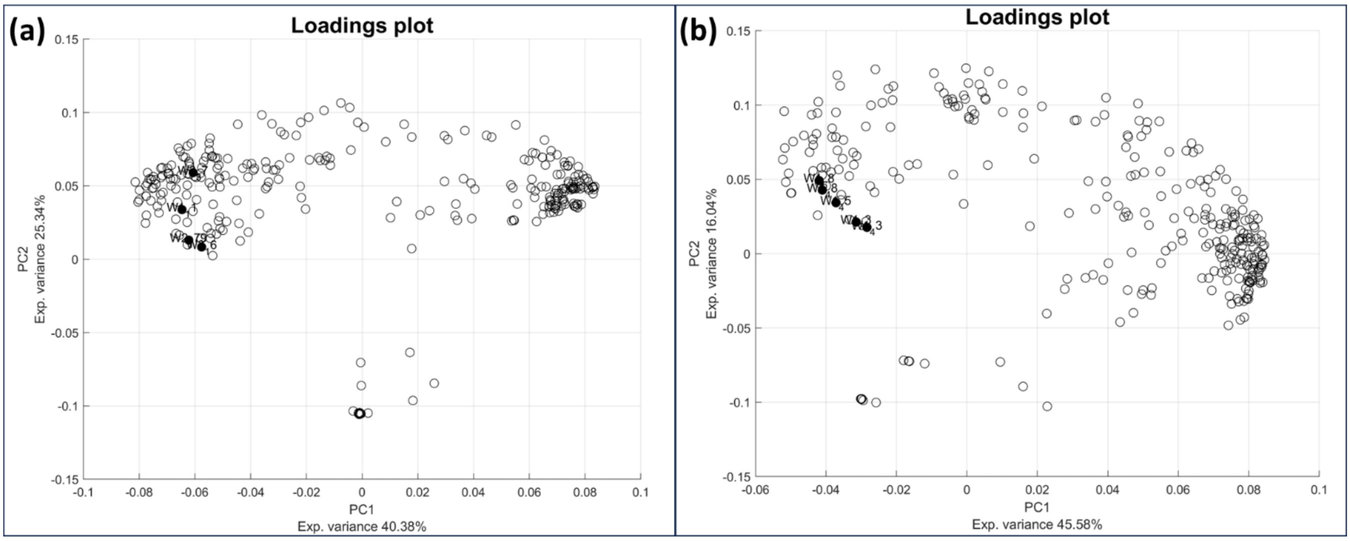

Figure S11: *Loadings plots on the first two principal components for the data obtained by positive ionization mode(a) and negative ionization mode (b). The input for the PCA was the matrix including the peak area of all resolved MCR components of the models for all samples and standards.*
